# HROB Is Implicated in DNA Replication

**DOI:** 10.3390/genes15121587

**Published:** 2024-12-10

**Authors:** Julia Kutz, Hannes Schmietendorf, Sheikh Anika Rahman, Franz Opel, Helmut Pospiech

**Affiliations:** 1 Project Group Biochemistry, Leibniz Institute on Aging—Fritz Lipmann Institute, D-07745 Jena, Germany; julia.kutz@leibniz-fli.de (J.K.); hannes.schmietendorf@gmx.de (H.S.); autushi222@gmail.com (S.A.R.); opel@boyle-institut.de (F.O.); 2 Institute of Biochemistry and Biophysics, Faculty of Biological Sciences, Friedrich Schiller University, D-07745 Jena, Germany; 3 Department of Medical Engineering and Biotechnology, Ernst-Abbe University of Applied Sciences, D-07745 Jena, Germany; 4 Department of Obstetrics and Gynecology, University Hospital Düsseldorf and Heinrich-Heine University, D-40225 Düsseldorf, Germany

**Keywords:** DNA replication, HROB, DNA interstrand crosslink (ICL) repair, Förster resonance energy transfer (FRET), protein overexpression, soluble protein fragment identification

## Abstract

DNA replication represents a series of precisely regulated events performed by a complex protein machinery that guarantees accurate duplication of the genetic information. Since DNA replication is permanently faced by a variety of exogenous and endogenous stressors, DNA damage response, repair and replication must be closely coordinated to maintain genomic integrity. HROB has been identified recently as a binding partner and activator of the Mcm8/9 helicase involved in DNA interstrand crosslink (ICL) repair. We identified HROB independently as a nuclear protein whose expression is co-regulated with various DNA replication factors. Accordingly, the HROB protein level showed a maximum in S phase and a downregulation in quiescence. Structural prediction and homology searches revealed that HROB is a largely intrinsically disordered protein bearing a helix-rich region and a canonical oligonucleotide/oligosaccharide-binding-fold motif that originated early in eukaryotic evolution. Employing a flow cytometry Förster resonance energy transfer (FRET) assay, we detected associations between HROB and proteins of the DNA replication machinery. Moreover, ectopic expression of HROB protein led to an almost complete shutdown of DNA replication. The available data imply a function for HROB during DNA replication across barriers such as ICLs.

## 1. Introduction

The correct and complete duplication of the genetic information in each cell cycle is crucial for maintenance of genome stability in all organisms. Errors in DNA replication lead to the accumulation of mutations, chromosomal aberrations and genomic instability and are thus associated with disruption of cellular homeostasis, tumorigenesis and aging [1,2,3]. To ensure the maintenance of genetic integrity, eukaryotic DNA replication occurs as a coordinated interplay of many proteins whose assembly and activation are subject to strict regulatory mechanisms [4,5,6].

The initiation of DNA replication starts in the early G1 phase of the cell cycle by the loading of Mcm2-7 (minichromosome maintenance) complexes on the origins of replication in a process called licensing. Restriction of replication licensing to the G1 phase guarantees that the genome is replicated once and only once every cell cycle [5,6,7].

The hexameric Mcm2-7 complex represents the catalytical core of the replicative DNA helicase [8]. It is kept in an inactive state until activation of specific origins during S phase. Here, a complex entailing RecQL4, Treslin and TopBP1 will promote concurrent opening of the DNA and the conversion of Mcm2-7 into the active DNA helicase by recruiting Cdc45 (cell division cycle) and the GINS (Japanese go-ichi-ni-san, five-one-two-three) complex, consisting of Sld5 and Psf1-3 [7,9,10,11,12]. This reaction is regulated by the concerted activity of the S phase-specific kinase complexes S-CDK (S phase cell division kinase) and DDK (dumbbell former 4-dependent cell division cycle 7 kinase) [4,5,6,7,13,14,15,16]. After attaching to the leading strand template DNA polymerase ε and several other replication elongation factors, the replication fork is established. Thereafter, the unwinding of the DNA continues and two replication forks progress in opposite directions. RPA (replication protein A) stabilizes the resulting single-stranded DNA (ssDNA) [4,7].

While approximately 250,000 potential replication start sites are licensed during G1, only 30,000–50,000 are used to initiate DNA synthesis in the S phase [17,18,19,20]. These additional start points, known as dormant replication origins, serve as reserves that may be activated within the DNA damage response. In this way, they fulfill an important function in ensuring complete replication of the genome [21,22]. This back-up mechanism is necessary because DNA replication is exposed to a variety of exogenous and endogenous stressors resulting in slowed or stalled replication fork progression, DNA breaks and thus reduced replication fidelity [3,5].

During elongation, replication forks encounter barriers such as DNA lesions, DNA repair intermediates, secondary DNA structures and transcription–replication conflicts. Therefore, DNA repair and DNA replication must be closely coordinated to protect genomic integrity. Homologous recombination (HR) guarantees the completion of DNA replication by facilitating the repair and reactivation of stalled replication forks, replication fork remodeling and protection and the repair of post-replicative gaps [23,24,25].

Mcm8 and Mcm9 are homologs of Mcm2-7. Similarly to the latter, Mcm8 and Mcm9 form a heterohexameric helicase complex whose function is not fully understood yet [26,27,28,29,30,31]. While they were initially thought to be mainly involved in DNA replication based on localization at replication foci and association with DNA replication factors [32,33,34,35], more recent studies suggest a function of the helicase in HR-associated DNA synthesis [28,30,31,36,37,38].

The latter is based on the observation that Mcm8 or Mcm9 deficiency leads to genomic instability, a strong hypersensitivity to DNA crosslinking agents such as cisplatin and mitomycin C, and causes a reduction in HR repair efficiency [28,39,40,41,42]. The Mcm8/9 helicase is required for gametogenesis and tumor suppression in mice and mutations in the *MCM8* and *MCM9* genes are associated with premature ovarian failure in women [37,43,44,45]. Consistent with their requirement for resistance against DNA interstrand crosslinks (ICLs), Mcm8/9 associates with ICL-induced damage foci wherein Mcm9 directly interacts with Rad51. It has therefore been assumed that the Mcm8/9 helicase enables the extension of the invading strand in D-loops if extensive DNA synthesis is required in the context of ICL repair [28,38,40,41,42].

On the other hand, cells lacking Mcm8/9 show a reduction in DNA replication also in the absence of DNA-damaging agents, accompanied by a strong increase in stalled or collapsed forks and genomic instability. This implicates Mcm8/9 rather in replication-coupled repair and makes it thus relevant for stabilization and protection of forks during normal DNA replication [34,35,37,45,46,47].

HROB (also known as Mcm8IP and C17orf53) has been identified by several groups as an interaction partner of the Mcm8/9 helicase involved in HR [31,36,48]. Originally proposed as a helicase loading factor [36], more recent data implicate HROB rather as an activator that stimulates translocation and unwinding activity of the Mcm8/9 helicase [49]. We identified HROB independently of these works as a highly conserved nuclear protein showing a shared expression regulation with various DNA replication factors. Based on this, we characterized HROB and confirmed the S-phase-specific and proliferation-associated expression and revealed an association with components of the replisome. Furthermore, a strongly elevated cellular HROB protein level dramatically interfered with DNA replication. Combining these findings with its structural properties and previously published results, we suggest a role for HROB during DNA replication across unsurmountable barriers such as ICLs.

## 2. Materials and Methods

### 2.1. Cloning and Plasmids

The plasmid pcDNA3.1(+) was obtained by Invitrogen (Carlsbad, CA, USA). The plasmids pcDNA3-mCer, pcDNA3-mVen and pcDNA3-mVen-mCer were generous gifts from Dr. Sakari Kellokumpu (University of Oulu, Oulu, Finland). For the preparation of plasmids coding for Cerulean or Venus fusion proteins, the complete open reading frame of the relevant human protein was amplified using primers bearing restriction sites. All primers were obtained from Eurofins Genomics (Ebersberg, Germany) and are listed in Supplementary Table S1. The resulting PCR product was cloned into the target plasmids by conventional restriction with two suitable enzymes. The restriction enzymes used were from Thermo Fisher Scientific (Rockford, IL, USA). For the generation of C-terminally tagged fusion proteins, pcDNA3-mCer or pcDNA3-mVen were used, and for N-terminally tagged fusion proteins, the PCR product was cloned into pmCerulean-C1 or pmVenus-C1 (Addgene, Watertown, MA, USA, #27796 and #27794, [50]), respectively. For the preparation of pRSF-Duet-6His-C17orf53 used for protein purification of HROB, the PCR product was inserted into pRSFDuet-1 obtained from Novagen (EMD Millipore, Carrigtwohill, Ireland). All plasmids listed in Supplementary Table S1 were deposited at Addgene.

The shRNA-containing plasmids (pGIPZ non-silencing shRNA control, RHS4346; pGIPZ C17orf53 shRNA V3LHS_325872: TCCACAGCAAACAGCTTCT, V3LHS_325874: TTTAGCCAATACTGAACCC, V3LHS_325875: TGAACAGGGGAGCGTTGCG; RHS4531-EG78995) used for the generation of knock-down cell lines were obtained from Horizon Discovery (Cambridge, UK).

For the random PCR screen, the plasmid pEGGsH6 [51] was first optimized. To create the plasmid pEGGsH6-MCS_SHORT_inFrame, the hybridized cloning oligos 5′-CGACATATGCGCGGCCGCAGATCTG-3′ and 5′-CTAGCAGATCTGCGGCCGCGCATATGT-3′ were inserted into the *Xmi*I and *Nhe*I sites of pEGGsH6. On the one hand, this shortened the multiple cloning site (MCS), which also reduced the length of the amino acid sequence between the glutathione-*S*-transferase and the analyzed protein during the screen. At the same time, the number of charged amino acids was reduced. On the other hand, an additional *Not*I site was inserted. The plasmid pEGGsH6-MCS_SHORT_outFrame was created similarly by insertion of the hybridized cloning oligos 5′-CGACATATGCGCGGCCGCAGATCTTGG-3′ and 5′-CTAGCCAAGATCTGCGGCCGCGCATATGT-3′ into the *Xmi*I and *Nhe*I sites of pEGGsH6. Here, the MCS was constructed in a way that the downstream GFP gene was out of the open reading frame. This guarantees that re-ligation events without integration of sequence fragments do not lead to false positive green fluorescent colonies. Both plasmids were deposited to Addgene (#211438 and #211439).

The complete open reading frame of all used plasmids was verified by Sanger sequencing before use. Plasmid purification was performed using the NucleoBond PC 100 Midi kit (Macherey-Nagel, Düren, Germany, 740573).

### 2.2. Random PCR Screen

For identification of soluble N-terminal fragments of HROB, the random PCR screen as described by [51] was modified and adapted to HROB.

The optimized screening plasmid pEGGsH6-MCS_SHORT_outFrame was mainly used for the assay. Additionally, four fragments were identified using pEGGsH6-MCS_SHORT_inFrame, which was used as a control in initial experiments with the optimized plasmids. Therefore, the forward primers Primer A A_HROB_*Nde*I_fwd (5′-GTACTCCGACAGCATATGGATGGCGTGCAGTTTGCAG-3′) and Primer B B_*Nde*I_fwd (5′-GTACTCCGACAGCATATG-3′) and the reverse primers Primer C C_random_*Bgl*II_fwd (5′-TACCCGCAGCGTAGATCTNNNNNNNNNNNNNNN-3′) and Primer D D_*Bgl*II_rev (5′-TACCCGCAGCGTAGATCT-3′) were used. Initially, the screening was performed using pEGGsH6. Here, the following primers were used. Primer A: A_HROB_*Xmi*I_fdw (5′-GTACTCCGACAGGTCGACATGGCGTGCAGTTTGCAG-3′); Primer B: B_*Xmi*I_fwd (5′-GTACTCCGACAGGTCGAC-3′); Primer C: C_random_*Xho*I_rev (5′-TACCCGCAGCGTCTCGAGNNNNNNNNNNNNNNN-3′); Primer D: D_*Xho*I_rev (5′-TACCCGCAGCGTCTCGAG-3′).

The PCR was performed in 50 µL reactions with Phusion DNA polymerase and GC buffer (Thermo Fisher Scientific). After 15 cycles of 15 s at 98 °C, 30 s at 60 °C and 45 s at 72 °C in the presence of 20 µM forward primer Primer A and 5 ng template, the reverse primer Primer C was added at 20 µM, followed by 5 cycles of 30 s at 98 °C, and 60 s at 80, 70, 60, 50, 40 and finally at 72 °C. PCR products were purified with the GeneJET PCR purification kit (Thermo Fisher Scientific) and amplified with primers B and D using Phusion DNA polymerase with GC buffer and 40 cycles of 10 s at 98 °C, 30 s at 60 °C and 45 s at 72 °C. PCR products were visible as a smear without separate bands after agarose gel electrophoresis. Products corresponding to 250–1250 and 1250–2000 bp were excised separately and purified using the MinElute DNA extraction kit (Qiagen, Hilden, Germany). After digestion with *Xmi*I and *Xho*I or *Nde*I and *Bgl*II (Thermo Fisher Scientific), respectively, the fragment library was ligated into the screening vector and plated on lysogeny broth (LB)*/*ampicillin (100 μg/mL, Sigma-Alderich, Steinheim, Germany) plates. Colonies displaying fluorescence were cultured and plasmids were purified using the GeneJET Plasmid Miniprep Kit (Thermo Fisher Scientific). Plasmids were sequenced if inserts were detected in analytical PCR performed in 25 µL reactions using Taq DNA polymerase and buffer B (Bio&SELL, Feucht, Germany) with 20 µM pGEX-5-fwd (5′-GGGCTGGCAAGCCACGTTTGGTG-3′) and EGFP_N-rev (5′-CGTCGCCGTCCAGCTCGACCAG-3′). Data analysis was performed using Chromas Lite 2.01 (Technelysium, South Brisbane, Australia) and the NCBI (National Center for Biotechnology Information) blastN suite [52].

### 2.3. Cell Culture, Induction of Quiescence, Compound Treatment and Transfection

HEp-2 (ATCC: CCL-23), U2OS (ATCC: HTB-96) and BJ (ATCC: CRL-2522) cells were cultivated in Dulbecco’s Modified Eagle’s Medium (DMEM, PAN Biotech GmbH, Aidenbach, Germany, E15-877) supplemented with 10% fetal bovine serum (FBS, PAN Biotech GmbH, P30-3306), 1.8 mM L-glutamine (Sigma-Aldrich, G7513-100ML) and 0.9 mM sodium pyruvate (PAN Biotech GmbH, P04-43100) at 37 °C, with 5% CO_2_ and 95% relative humidity.

To induce quiescence, BJ cells were cultured for at least four weeks without passaging in DMEM with an addition of 0.5% FBS. The medium was changed every 2–3 days. Untreated BJ cells with passage < 20 were used as non-quiescent (proliferating) control cells, which were regularly passaged and cultured in DMEM containing 10% FBS.

For synchronization, cells were treated for 18 h with 9 µM of the CDK1 inhibitor RO-3306 (Sigma-Aldrich, SML0569-25MG), followed by release into full medium for the indicated time.

To prepare control samples with apoptotic cells, cells were cultured for 18–23 h in DMEM supplemented with 10 µM Actinomycin D (Enzo Life Sciences, Lörrach, Germany, BML-GR300).

Transfection was performed using the electroporator Nucleofector II (Amaxa Biosystems, Gaithersburg, MD, USA) with the program O-017 for HEp-2 cells or X-001 for U2OS cells, respectively. A total of 50–70% confluent cells were trypsinized (0.25% trypsin (c.c.pro, Oberdorla, Germany, Z-25-M), 9 mM EDTA (Carl Roth, Karlsruhe, Germany, 8040.2) in PBS (Sigma-Aldrich, D8537)), washed in DMEM, counted (cell counter Cellometer Auto T4, Nexcelom Bioscience, Manchester, UK), and washed with PBS. For each transfection, 1 million cells were suspended in 95 µL electroporation solution (Ingenio, Mirus Bio, Madison, WI, USA, MIR 50117) containing 3 µg of each of the desired plasmids and transferred into an electroporation cuvette (Ingenio, 0.2 cm, Mirus Bio, MIR 50121). After electroporation, the suspension was transferred to a cell culture dish with DMEM and incubated for 16–20 h. For the generation of HEp-2 cells stably transfected with pGIPZ plasmids, beginning from 24 h after electroporation, the cells were continuously cultured in DMEM with an addition of 2 μg/mL puromycin (InvivoGen, Toulouse, France, ant-pr-1).

### 2.4. Flow Cytometry

For detection of DNA synthesis, 20 min before harvesting cells, 5-ethynyl-2′-deoxy-uridine (EdU, Jena Bioscience, Jena, Germany, CLK-N001) was added to a final concentration of 10 µM. Cells were trypsinized, washed with PBS (Sigma-Aldrich), fixed with 2% formaldehyde (Carl Roth, #4980) in PBS for 10 min at room temperature and permeabilized with 0.1% Triton X-100 (Carl Roth) in PBS for 5 min at room temperature. Click-chemistry-based azide coupling for EdU detection was performed by incubation for 30 min with 0.88 μg/mL Alexa Fluor 647 azide (Invitrogen, A10277) in PBS with 2.5 mM CuSO_4_ (Merck KGaA, Darmstadt, Germany) and 49.5 mM freshly prepared sodium L-ascorbate (Sigma-Aldrich).

To stain the DNA, 4,6-Diamidino-2-phenylindole (DAPI, Sigma-Aldrich) was added to permeabilized samples to a final concentration of 4.3 µM immediately before analysis. The CellTrace Violet kit (Invitrogen, C34557) was used for the cytometry-based determination of the growth rate according to manufacturer’s instructions.

**Figure 1:**
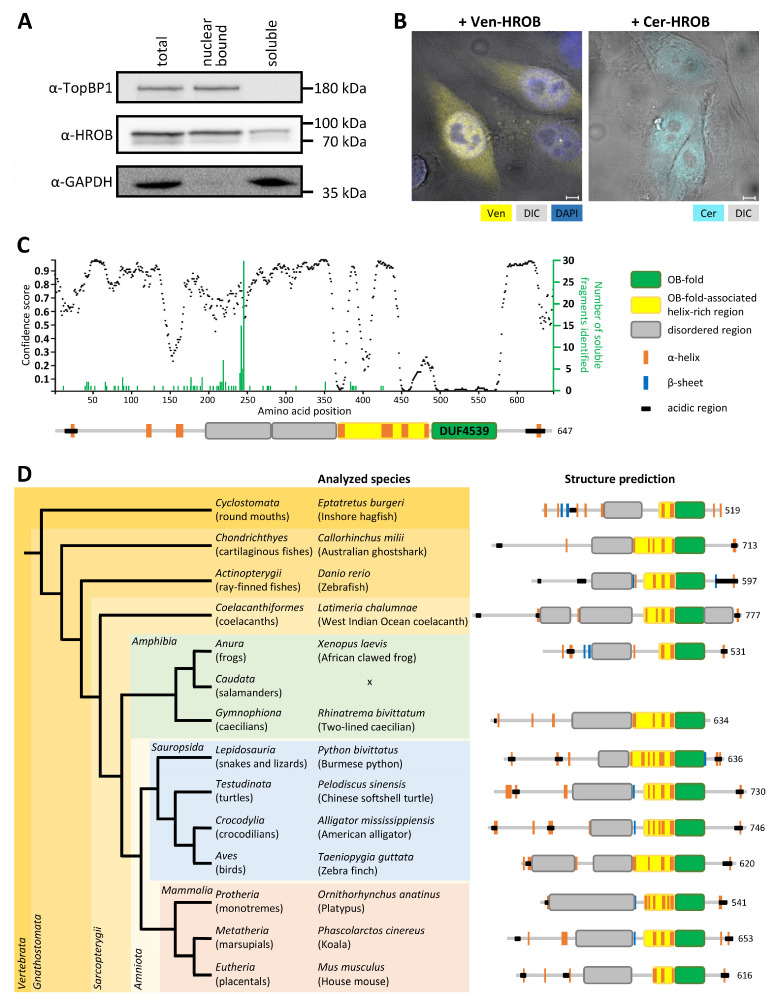
HROB is a largely disordered conserved nuclear protein bearing an OB-fold. (**A**) Western blot of total extracts and subcellular fractionated extracts of HEp-2 cells was performed using antibodies recognizing the indicated proteins. TopBP1 and GAPDH were used as a control for an exclusive nuclear and an exclusive cytosolic protein, respectively (abbreviation: α—anti, n = 2). (**B)** Fluorescence microscopy of transiently transfected HEp-2 cells expressing HROB fusion proteins with Venus (Ven, yellow; co-stained with DAPI, blue) or Cerulean (Cer, turquoise). Cell morphology was captured using differential interference contrast (DIC, grey). Scale bar: 5 µm (n = 2). (**C**) (**top**) The intrinsic disorder prediction was created using DISOPRED (version 3) [56]. The number of soluble fragments of different lengths identified using the random PCR screen in eight independent experiments are indicated in green. (**bottom**) Scheme of the human HROB structure prediction performed with PSIPRED (version 4.0) [57]. The OB-fold (oligonucleotide/oligosaccharide-binding fold motif, domain of unknown function 4539, N493-D576, green) was identified with HMMER (version 3.3) HMMSCAN using Pfam [58]. α-Helices (orange) and β-sheets (blue) outside of the OB-fold were shown from a minimum length of five amino acids. OB-fold-associated helix-rich regions (yellow) were defined as sections with a proportion of at least 40% helices (independent of the length). Disordered regions (gray) were defined as at least 80 amino acid long, undisrupted sections lacking any α-helix or β-sheet predictions. Acidic regions (black) were identified using SAPS [59] with manual post-selection of regions with at least seven and at least 30% of negatively charged amino acids and without any positively charged amino acid. (**D**) HROB homologs were harvested by PSI-BLAST searches in Ensembl (version 99) and NCBI protein databanks [60,61], as well as from Pfam (version 32.0) [62]. Structure prediction and scheme generation was performed as described for human HROB in (C). The numbers indicate the number of amino acids of the proteins. For taxonomic classification, NCBI Taxonomy (version 12/2019) [63] and Open Tree of Life (version 3.2) [64] were used. The protein-IDs of all shown homologs are listed in Supplementary Table S2 (illustration inspired by [65]).

For the cytometry Förster resonance energy transfer (FRET) assay, the transfected cells were trypsinized 18–20 h after transfection and resuspended in DMEM without phenol red (PAA Laboratories, Cölbe, Germany, E15-877). Analysis of living cells was performed immediately afterwards (see also Supplementary Figure S9). We applied the flow cytometry FRET assay to evaluate association of HROB with proteins of the DNA replication machinery. We utilized transient ectopic expression of replication factors fused to the fluorescent proteins Cerulean (Cer) and Venus (Ven), which are particularly well suited for flow cytometry FRET analyses [53,54,55]. To limit overexpression, analysis was performed one day after transient expression with the respective expression vectors, and the correct expression and nuclear localization of the fusion proteins was verified by Western blot and fluorescence microscopy of transfected cells, respectively (Supplementary Figures S7 and S8 and Figure 1B).

Every experiment included samples co-expressing the two protein fusions of interest as well as a FRET-positive control expressing a Ven-Cer fusion protein and FRET-negative controls. These expressed either Cer or Ven as a fusion with the protein of interest, whereas the other fluorescent protein was unfused, to exclude an interaction of the free fluorescence protein with the protein of interest (see Figure 4A and Supplementary Figure S11A below). While FRET could be detected in 95–99% of the cells in the FRET-positive control samples, the FRET-negative controls with cells expressing a fusion protein and a free fluorescent protein did not show FRET in any of the experiments performed (see Supplementary Figures S9 and S11A). The functionality of the experimental conditions and the selected controls was confirmed using the well-characterized interaction of Cdc45 and Mcm5 [9,10,11,12] (see Supplementary Figure S11A–C).

Flow cytometry was performed with a BD FACSVerse flow cytometer (BD Biosciences, Heidelberg, Germany) equipped with three-laser configuration (violet, blue and red), using the FACSSuite software (1.0.5.3841; BD Biosciences, Franklin Lakes, NJ, USA), and data were subsequently analyzed using FlowJo (v7.6.5; Tree Star, Ashland, OR, USA). The utilized gating schemes are shown in Supplementary Figure S10. Final analyses were performed using Excel 2016 (Microsoft, Redmond, WA, USA). Significances were determined using Student’s *t*-tests (* *p* < 0.05, ** *p* < 0.01, *** *p* < 0.001) without adjustment for multiple testing.

### 2.5. Preparation of Cell Extracts, Subcellular Fractionation and Western Blot

For preparation of cell extracts for Western analysis, cells were washed once with PBS and lysed with RIPA buffer (150 mM NaCl, 1 mM Na_2_EDTA, 1 mM EGTA, 1% NP-40, 1% sodium deoxycholate, 2.5 mM sodium pyrophosphate, 0.1% SDS, 20 mM Tris-HCl, pH 7.5) supplemented with protease and phosphatase inhibitors (1× cOmplete EDTA-free; PhosSTOP Easy pack, Roche, Mannheim, Germany), incubated on ice for 1 h, followed by sonication (Sonifier W-450D, Branson Ultrasonics, Danbury, CT, USA) for 30 s at 25% amplitude. If Cdc45 and Mcm5 were detected in Western blot, NETN300 buffer (300 mM NaCl, 0.5 mM Na_2_EDTA, 0.5% NP-40, 20 mM Tris-HCl, pH 8.0) was used instead of RIPA. Laemmli buffer (final concentration, 1% SDS, 2.5% 2-mercaptoethanol, 10% glycerol, 0.0005% bromophenol blue, 25 mM Tris-HCl, pH 6.8) was then added.

To obtain subcellular fractionated cell extracts, cells were trypsinized, resuspended in PBS and counted as indicated in Section 2.3. As a full extract control, the pellet of 2 million cells was lysed in 300 μL double-concentrated Laemmli buffer (final concentration, 2% SDS, 5% β-mercaptoethanol, 20% glycerol, 0.001% bromophenol blue, 50 mM Tris-HCl, pH 6.8) and sonicated for 30 s at 25% amplitude (Sonifier W-450D, Branson Ultrasonics). The preparation of the soluble and nuclear bound fractions was performed as described previously [66]. The pellet of 2 million cells was resuspended in 400 μL fractionation lysis buffer (10 mM HEPES-KOH, 10 mM KCl, 3 mM Na_2_EDTA, 0.34 M saccharose, 10% glycerol, 1 mM dithiothreitol, 0.1 mM phenylmethylsulfonyl fluoride) supplemented with protease and phosphatase inhibitors (1× cOmplete EDTA-free; PhosSTOP Easy pack, Roche). After the addition of Triton X-100 to a final concentration of 0.125%, samples were incubated on ice for 5 min and centrifuged at 4 °C for 4 min at 1300× *g*. The supernatant representing the soluble fraction was cleared at 4 °C for 15 min at 20,000× *g* and mixed with Laemmli buffer. For the nuclear bound fraction, the pellet was washed two times with fractionation lysis buffer via centrifugation at 4 °C for 4 min at 1300× *g*, lysed in 200 µL double-concentrated Laemmli buffer and sonicated for 30 s at 25% amplitude (Sonifier W-450D, Branson Ultrasonics).

Protein extracts of 100,000 cells (125,000 cells for subcellular fractionated cell extracts) were separated by SDS-PAGE in 7.5%, 10% or 12.5% gels and transferred onto an Immobilon-P PVDF membrane (0.45 µm pores, Merck Millipore, Carrigtwohill, Ireland, #IPVH00010). Membranes were blocked for 1 h with 4% skim milk in Tris-buffered saline (TBS, 137 mM NaCl, 2.7 mM KCl, 25 mM Tris-HCl, pH 7.4) and probed with primary antibodies overnight at 4 °C (Cell Signaling technology (Danvers, MA, USA): anti-GFP (2555), anti-PARP1 (poly [ADP-ribose] polymerase 1, 9542); Merck Millipore (Schwalbach am Taunus, Germany): anti-γH2AX-S139 (H2A histone family member X serin 139, JBW301); Proteintech (Manchester, UK): anti-Mcm5 (11703-1-AP); Santa Cruz Biotechnology (Dallas, TX, USA): anti-cyclin A (sc-751), anti-GAPDH (glyceraldehyde 3-phosphate dehydrogenase, sc-32233), anti-HSC70-HRP (heat shock cognate 71 kDa protein, sc-7298), anti-Mcm2 (sc-9839); Sigma-Aldrich: anti-β-actin (A5441), anti-HROB (HPA023392); Thermo Fisher Scientific: anti-PCNA (proliferating cell nuclear antigen, MA5-11358), non-commercial antibodies: rat monoclonal anti-Cdc45 (3G10 [67]), rabbit polyclonal anti-RecQL4 [68], mouse monoclonal anti-RPA32 (34A [69]), rabbit polyclonal anti-TopBP1 (TopBP1.2 [70])). The Greek letter α was used as the abbreviation of “anti” in the labeling of the Western blot-containing figures. Primary antibodies were detected with horseradish peroxidase (HRP)- or alkalic phosphatase (AP)-conjugated secondary antibodies (Promega (Madison, WI, USA): anti-goat-IgG-HRP, anti-mouse-IgG-HRP/AP, anti-rabbit-IgG-HRP/AP; Jackson ImmunoResearch (Ely, UK): anti-rat-IgG-HRP). Immunoblots were developed with SuperSignal West Pico/Femto Chemiluminescent Substrate (Thermo Fisher Scientific) for HRP and Immun-Star (Bio-Rad Laboratories, Hercules, CA, USA) for AP as described by the manufacturer, using a gel documentation device (G:BOX F3, Syngene, VWR, Darmstadt, Germany). Signal intensity of Western blots was quantified using Image J (Wayne Rasband, National Institutes of Health, Bethesda, MD, USA, https://imagej.net/ij/, 1997–2018 (accessed on 3 June 2024)) and subsequent analysis was performed using Excel 2016 (Microsoft).

### 2.6. Bioinformatic Analyses of HROB and Its Homologues

Secondary structure predictions were performed using PSIPRED [57] (see Supplementary Figure S2 and scheme in Figure 1C) and intrinsic disorder prediction using DISOPRED [56] (Figure 1C). Homology searches to identify functional domains employed HMMER [58] and Pfam [62]. In addition, homologous sequences were identified by BlastP [60] at NCBI and Ensembl [61].

## 3. Results

### 3.1. The Expression of Human HROB and of DNA Replication Proteins Is Co-Regulated

Although the core components of human DNA replication have been characterized, the role of several auxiliary and regulating factors still remains unclear, and it can be assumed that many of these factors are yet to be identified. To search for potential new factors involved in DNA replication, we used the GeneNetwork service provided by the University of Groningen [71], which utilizes human expression data to identify gene networks that display co-regulation under a large range of cell models and conditions. In this way, *HROB* was revealed as a factor with a common regulation with many DNA replication factors and other genes involved in DNA metabolism. In particular, *HROB* showed a strong co-expression with *CDC45*, *CDT1*, *MCM10*, *ORC1*, *GINS3* (Psf3) and *TICRR* (Treslin) and a weak yet significant co-expression with several other replication factors such as *GINS1/2* (Psf1/2), *RECQL4*, *MCM3* or *POLE* (DNA polymerase ε catalytic subunit) (Supplementary Figure S1A). There were other genes with very strong common positive regulation not directly implicated in DNA replication. These include *EXO1* (exonuclease 1) and *AUNIP* (aurora kinase A and ninein interacting protein), both involved in DNA repair by HR [72,73]. Further examples are the cyclin B1- and cyclin D1-regulating transcription factors *FOXM1* [74] and *MYBL2* [75], respectively, as well as *TROAP* (trophinin-associated protein), which is involved in the assembly of the spindle apparatus [76], and *PLK1*, the gene coding for the mitosis-regulating polo-like kinase 1 [77]. Based on this, GeneNetwork predicted a possible function of HROB in DNA replication, DNA repair, cell cycle and mitosis (Supplementary Figure S1). The *p* values given for a function in human DNA replication are between 10^−11^ and 10^−18^, depending on the signaling pathway database used, and thus comparable to the *p* values calculated for GeneNetwork searches conducted with DNA replication factors such as TopBP1 or RecQL4 (Supplementary Figure S1B).

In accordance with the functional prediction, a nuclear localization of HROB could be confirmed. Subcellular fractionation experiments showed that the protein is mostly present in the nuclear bound fraction, and fluorescent HROB fusion proteins also showed a strong, albeit not complete, overlap of fluorescence with DNA staining, suggesting together a chromatin association of HROB (Figure 1A,B).

### 3.2. HROB Is Conserved Within Eukaryotes

HROB has been identified as an OB-fold domain-containing protein. This OB-fold has been recently implied to mediate its interaction with Mcm9 [36,49]. Outside that domain, HROB is predicted to be largely disordered (Figure 1C; see also e.g., AlphaFold prediction at UniProt, https://www.uniprot.org/uniprotkb/Q8N3J3/entry (accessed on 14 October 2024)).

**Figure 2:**
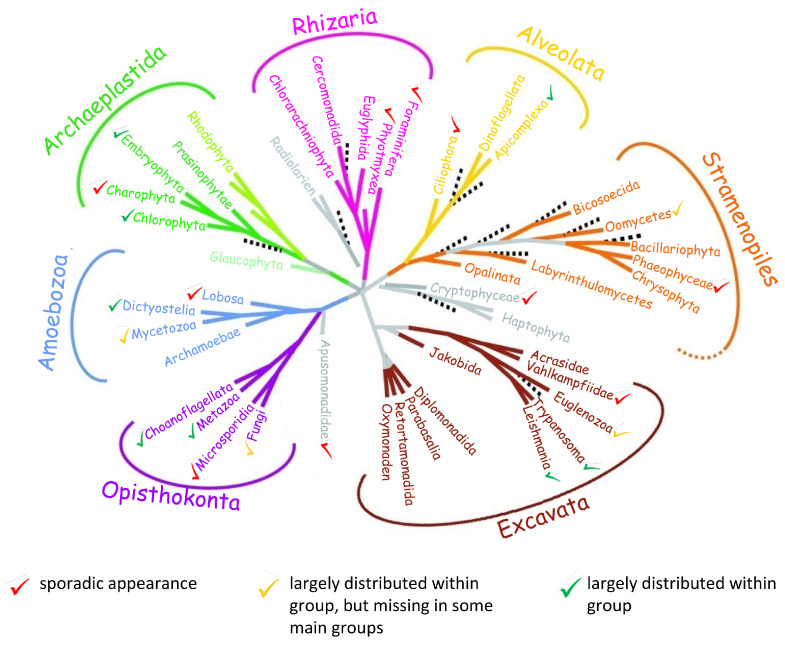
HROB homologs were identified in all main groups of eukaryotes. Identification of HROB sequence homologs was performed as described in Figure 1 and Section 2. Taxonomic position of *Microsporidia* is based on [78]. Groups labeled with green check marks show a wide distribution of HROB. In groups labeled with yellow check marks, homologues are also widely distributed but could not be identified in some main groups. A sporadic occurrence of HROB homologs is marked with red check marks (illustration is inspired by [79]).

To obtain a better insight into the phylogenic origin of conserved structural properties and features of HROB and its homologues, we performed extensive harvesting of HROB homologues from different organisms followed by bioinformatics analysis of HROB homologues from representative species. In vertebrates, HROB displays a highly conserved structural organization despite the low levels of sequence homology outside the OB-domain and large variation in protein size between different organisms (Figure 1C,D). Structural features besides the OB-fold domain (identified as a Pfam domain of unknown function 4539 (DUF4539)) include a carboxy-proximal, helix-rich OB-associated region. Other conserved features are the intrinsically disordered region immediately preceding the extended OB-fold, regions with very limited secondary structure in the N-terminus and the C-terminus of HROB as well as the presence of sequence patches rich in acidic residues close to both ends of the protein sequence (Figure 1C,D and Supplementary Figure S2). HROB homologues are also widely distributed within the eukaryotic domain but essentially absent in Eubacteria and Archaea (Figure 2). However, groups of eukaryotes differ considerably regarding the distribution of HROB. Some kingdoms (e.g., *Metazoa*, *Embryophyta*) show a very broad distribution, and only in some suborders (such as ferns or flies), homologues of HROB were not identified. On the other hand, no homologues could be found in other divisions (e.g., red algae, diatoms). Even within vertebrates, some species such as chicken and the clade of salamanders do not appear to possess HROB homologues. These data suggest that *HROB* is an early eukaryotic gene that has been lost independently in several groups and species during evolution. Curiously, no orthologues of HROB could be identified in many model organisms such as *S. cerevisiae*, *C. elegans* and *D. melanogaster*. Structural predictions of HROB homologues of different eukaryotic clades display a higher variability regarding structural features outside the conserved OB-fold. Nevertheless, the helix-rich region directly upstream of the OB-fold (Figure 1C) could be detected in all homologues examined.

Additionally, the proteins often exhibit highly negatively charged patches and regions of low complexity (Supplementary Figure S3). Taken together, structural and phylogenetic analyses argue for a basic, but not essential, cellular function of HROB within the eukaryotic domain.

We sought to obtain additional information about the structural properties of HROB. Therefore, we utilized a random PCR screen approach [51] designed to identify soluble HROB fragments. A cDNA library representing fragments of variable lengths of the N-terminus of HROB was generated by semi-random PCR and inserted into a screening plasmid to be expressed amino-proximal to a GFP folding reporter. Fluorescence of the resulting *E. coli* colonies was observed when the inserted HROB fragment was in frame with GFP and when the translated protein was natively folded. To this end, 66 different soluble protein fragments of HROB were identified by sequencing (see green bar chart in Figure 1C and Supplementary Figure S4B). The distribution of fragments was not random but indicated an accumulation of fragments with specific lengths. In addition to fragments spanning from the N-terminus up to predicted domain or structure boundaries, there was a clear domain boundary within a protein segment predicted to be disordered (Figure 1C) with a prominent accumulation of fragment lengths between 240 and 244 amino acids (Supplementary Figure S4B). This argues for an autonomous function of the region comprising amino acids 1–240.

### 3.3. HROB Is a Cell Proliferation-Related Protein

**Figure 3:**
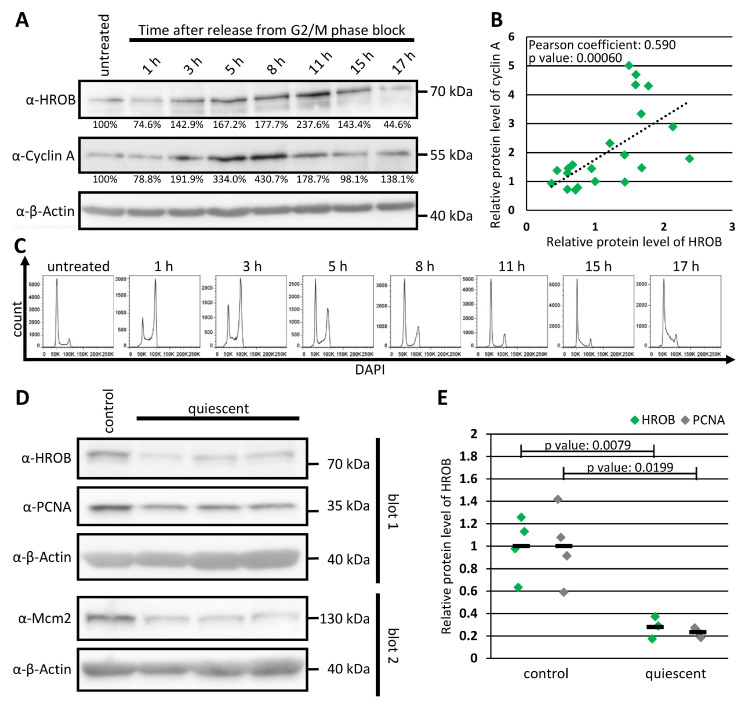
The cellular expression of HROB varies depending on cell cycle phase and proliferation state. (**A**–**C**) HEp-2 cells were treated for 18 h with the CDK1 inhibitor RO-3306. Untreated HEp-2 cells served as control. From 1 to 17 h after removal of the inhibitor, the cells were split, and the same samples were analyzed by both Western blot and flow cytometry (abbreviation: α—anti, n = 3). (**A**) Western blot of full cell extracts was performed using antibodies recognizing the indicated proteins. The percentages indicate the signal intensities of the shown HROB and Cyclin A protein bands to the β-actin loading control. Intensities are presented relative to the untreated (asynchronous) condition. (**B**) The normalized signal intensities of HROB and Cyclin A Western blot bands of three experiments show a strong correlation. Each green dot represents one sample and time point. The indicated *p* value was estimated by a classical two-sided Student’s *t*-test. (**C**) Verification of the synchronization of cells by DAPI staining and flow cytometric analysis. (**D**,**E**) For generation of quiescent cells, BJ cells were cultured for at least four weeks without passaging in medium with reduced FBS supplement (0.5%). Proliferating BJ cells served as control (n = 3–4). (**D**) Western blot of full cell extracts of control and quiescent BJ cells was performed using antibodies recognizing the indicated proteins. PCNA and Mcm2 served as control for proteins downregulated in quiescent cells (abbreviation: α—anti). (**E**) The signal intensities of the HROB and PCNA Western blot bands were quantified and corrected to the loading control (β-actin). The data were then normalized to achieve an average of one in the respective control groups. Each data point represents an independent biological sample. The means are shown as black bars. The indicated *p* values were determined by classical two-sided Student’s *t*-tests.

Cell cycle synchronization experiments indicated an S phase-specific upregulation of HROB expression (Figure 3A), as expected from the GeneNetwork analysis indicating co-expression with DNA replication factors (Supplementary Figure S1). HROB protein levels showed an upregulation simultaneous with the start of S phase, and there was a strong correlation between the expression of HROB and the S phase-specific cyclin A (Figure 3A–C). Using BJ primary human fibroblast cells, we also found that the HROB protein levels were reduced after induction of quiescence to an extent comparable to the proliferation marker PCNA (Figure 3D,E). This is in line with the cell-type-specific expression data in The Human Protein Atlas [80] (Supplementary Figure S5A) showing a strong HROB enrichment in mitotic cells.

Consistent with a proliferation-dependent expression, database analyses revealed HROB to be upregulated in many different cancer entities compared to the corresponding healthy tissue (Supplementary Figure S5B). In most cancer entities, a high HROB expression correlates with a poor prognosis (Supplementary Figure S6).

### 3.4. HROB Associates with DNA Replication Proteins

**Figure 4:**
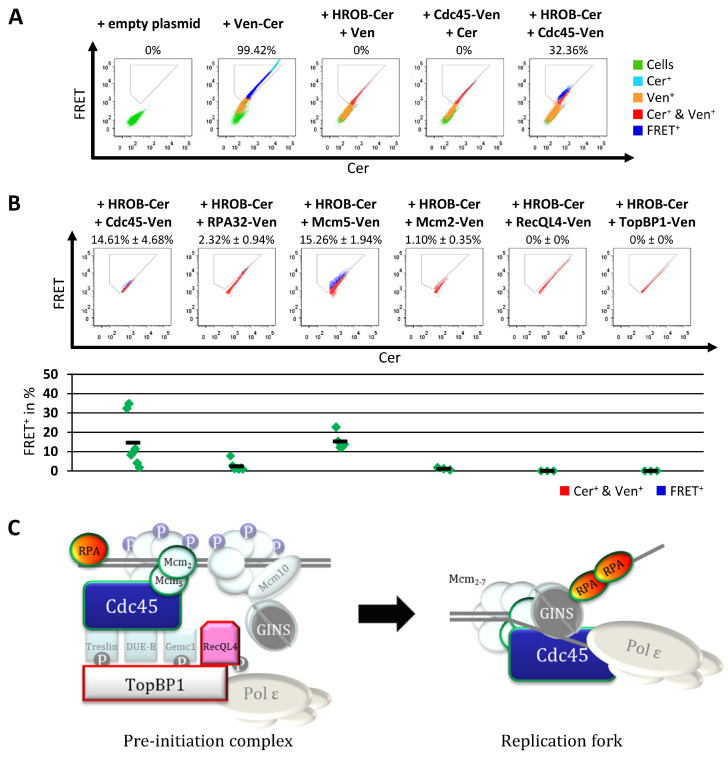
HROB shows FRET associations with DNA replication factors. HEp-2 cells were transiently transfected with empty vector or with plasmids encoding the indicated Cerulean (Cer) or Venus (Ven) fluorescent fusion proteins and analyzed by flow cytometry 18–20 h after transfection. (**A**) Representation of the sample set for each FRET experiment. Cells transfected with empty vector (pcDNA3.1+) served as a non-fluorescent control. Cells expressing the FRET-capable Ven-Cer fusion protein were used as positive control and cells transfected with not-fused Cer or Ven encoding plasmids in combination with a HROB or Cdc45 fluorescent fusion protein were used as negative controls. The intensities in the FRET channel were plotted against the Cer intensities. The populations are shown overlapping in the order cells (green), Cer-positive cells (Cer^+^, turquoise), Ven-positive cells (Ven^+^, orange), Cer- and Ven-double-positive cells (Cer^+^ and Ven^+^, red) and FRET-positive cells (FRET^+^, blue). The percentages indicate the respective proportion of FRET^+^ in the samples shown. (**B**) Representative images of samples of cells co-expressing HROB-Cer and Ven fusions of the indicated replication factors. The intensities in the FRET channel were plotted against the Cer intensities. The populations are shown overlapping in the order Cer- and Ven-double-positive cells (Cer^+^ and Ven^+^, red) and FRET-positive cells (FRET^+^, blue). The percentages indicate the mean (± SEM, standard error of the mean) of the proportions of FRET^+^ from at least three independent samples (n = 3–7, Supplementary Tables S4 and S5). (**C**) Schematic overview of the pre-initiation and elongation complex of the human DNA replication. Replication factors for which FRET to HROB was examined are highlighted. If FRET was detected between the factors and HROB, the factors are marked with a green outline; if not, the outline is colored red (illustration is inspired by [84]).

DNA replication represents a highly dynamic process characterized by many transient protein interactions. These are often mediated by the joint interplay of different proteins organized in complexes, wherefore detection using classical biochemical interaction assays is often limited. We therefore employed a FRET-based flow cytometry assay to probe for associations between HROB and various replication factors (Figure 4A and Supplementary Figures S7–S11). The functionality of the experimental conditions and the selected controls were confirmed using the well-characterized interaction of Cdc45 and Mcm5 (Supplementary Figure S11A–C and Supplementary Tables S4 and S6). In addition, we were repeatedly able to demonstrate a FRET association between Cdc45 and RecQL4 (Supplementary Figure S11D,E and Supplementary Tables S4 and S6), both implicated as part of the pre-initiation complex [5]. For initial systematic screening, HROB fused to a carboxy-proximal Cerulean fluorescent protein (HROB-Cer). FRET could not be detected between HROB and the replication initiation factors RecQL4 and TopBP1 in any of the samples examined (Figure 4B and Supplementary Tables S4 and S5). In contrast, HROB showed solid FRET association with Cdc45, Mcm2, Mcm5 and RPA, all involved in the elongation phase of DNA replication (Figure 4C). Taking the percentage of FRET-positive cells as an indicator, association of HROB with Cdc45 and Mcm5 is more pronounced compared to the association with Mcm2 or RPA. Importantly, the number of FRET-positive cells after co-expression of HROB with Cdc45 and Mcm5, respectively, is in the same range as the number of FRET-positive cells after co-expression of Cdc45 and Mcm5 (Figure 4B, Supplementary Figure S11B,C and Supplementary Tables S4–S6), two well-established factors that are part of the Cdc45-Mcm2-7-GINS (CMG) helicase that is in the core of the elongating replisome during S phase. Inspection of the flow cytometric results further indicates that the FRET association does not require high expression levels of HROB and its association partner, indicating that FRET is not driven by non-physiological overexpression of the fluorescent proteins (Figure 4A,B). It should anyway be noted that the level of FRET gives only a limited indication about the association or non-association of proteins, as the FRET efficiency is determined not only by the distance between the fluorophores but also by the orientation of the utilized fluorophores to each other. This becomes apparent when comparing different pairs of fusions probing the same FRET association. Furthermore, FRET indicates association irrespective of direct physical interaction, as long as proximity with a donor/acceptor distance is less than 10 nm [81,82,83]. Based on the consistent FRET association of HROB with multiple compounds of the CMG helicase, these results place HROB into close proximity to the elongating replisome.

### 3.5. HROB Overexpression Interferes with DNA Replication

The synthesis and degradation of many DNA replication factors are tightly regulated, as not only their cell-cycle-dependent presence is essential for DNA replication, but sub-processes are also regulated by the absolute amount of protein. Thus, some replication factors are limited and increased or decreased expression disrupts cell homeostasis [85,86,87,88,89]. While Mcm2-7 proteins are relatively abundant with a determined number of ~1,000,000 molecules/cell [90], a proliferating human cell only contains ~50,000–240,000 RPA molecules [69,91], ~100,000–120,000 molecules of Cdc6 [92] and for geminin, Cdt1 and Mcm10, a number of only ~30,000 molecules per cell was determined [93,94]. The number of Cdc45 molecules per cell determined at ~33,000–45,000 corresponds to the maximum number of replisomes simultaneously active in the S phase, which is why Cdc45 is regarded as limiting for human DNA replication [86,95,96].

Here, using recombinant protein and whole cell extracts of carefully determined cell numbers, the cellular number of HROB molecules was roughly estimated at around 100,000 HROB molecules per cell (106,200 ± 13,600 [± SEM], Supplementary Figure S12). This number is comparable to RPA and Cdc45 involved in elongation of DNA replication as well as Rad51 with an estimated number of 20,000–100,000 molecules per cell, which is considered limiting for HR [97].

To obtain more insights into the protein function of HROB, experiments aimed at manipulating its cellular protein level were performed. To this end, cell lines stably expressing shRNA against HROB were generated. Knock-down of HROB led to the reduction in cellular HROB protein level to approximately one-third compared to cells expressing a non-targeting control shRNA (Supplementary Figure S13A,B). However, no effect of this knock-down on the cell cycle progression and on the growth rate could be observed in these cells (Supplementary Figure S13C–F). Together with the results of the cell number determination, this indicates that HROB is not essential or at least not limiting for DNA replication and proliferation.

**Figure 5:**
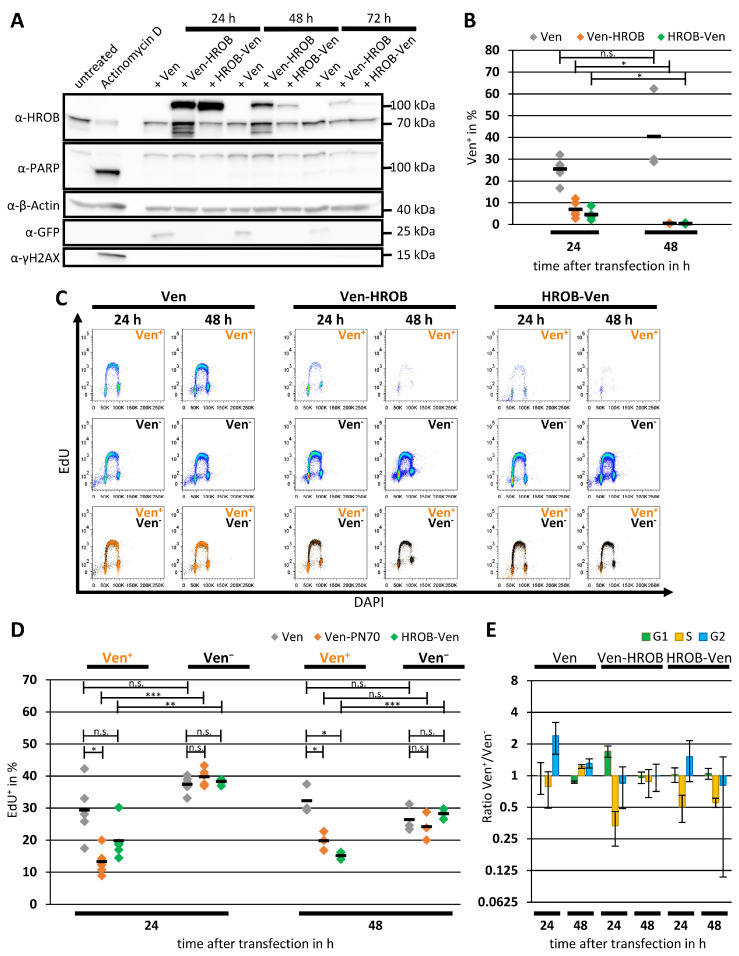
HROB-overexpressing cells show a depletion of S phase. HEp-2 cells were transiently transfected with plasmids encoding Venus (Ven), Ven-HROB or HROB-Ven. (**A**) Western blot was performed using full extracts of these cells 24, 48 or 72 h after transfection. Untreated cells or cells treated for 18–23 h with 10 µM Actinomycin D served as control (abbreviation: α—anti, n = 3). (**B**–**E**) A duration of 24 or 48 h after transfection, the cell cycle was analyzed via flow cytometry after EdU and DAPI staining. For each sample, Ven-positive (Ven^+^) and Ven-negative (Ven^−^) cells were distinguished, and the proportion of EdU-positive (EdU^+^) cells was determined (n = 3–5). (**B**) The cells were analyzed cytometrically for the proportion of Ven^+^ cells. Each data point represents an independent biological sample. The means are represented by black bars. *p* values (n.s.—not significant, * *p* < 0.05) were determined using two-sided Student’s *t*-tests without corrections for multiple testing. (**C**) Representative images of the cell cycle analysis diagrams. The EdU intensities were plotted against the DAPI intensities. The upper row shows Ven^+^ cells and the middle row Ven^−^ cells. Ven^+^ (in yellow) and Ven^−^ (in black) cells are overlayed within the diagrams of the bottom row. (**D**) The Ven^+^ and Ven^−^ cells were cytometrically analyzed for the proportion of EdU^+^. Each data point represents an independent biological sample. The means are represented by black bars. *p* values (n.s.—not significant, * *p* < 0.05, ** *p* < 0.01, *** *p* < 0.001) were determined using Student’s *t*-tests without corrections for multiple testing. (**E**) The cell cycle distribution of the Ven^+^ and Ven^−^ cells was determined. The means of the ratios of the values for the percentages of G1 (in green), S (in yellow) and G2 phase (in blue) are shown. The error bars indicate the standard deviations. The information on significance was omitted for reasons of clarity.

In contrast, strong effects on the cell cycle were detected when the HROB protein level was increased. Flow cytometric and Western analysis demonstrated a rapid loss of fluorescent cells after transient expression of HROB N- or C-terminally tagged to the fluorescent protein Ven over a period of three days (Figure 5A–C), whereas expression of Ven alone was well tolerated. The loss of HROB-overexpressing cells could not be attributed to apoptosis or DNA damage signaling, as only a trace signal of cleaved PARP and no accumulation of γH2AX could be detected (Figure 5A). The analysis of the cell cycle of the transfected cells also indicated that the observed decrease in Ven^+^ HROB-overexpressing cells was caused by reduced growth rather than apoptosis. Quantification of cells in S phase incorporating the thymidine analogue EdU after transient transfection demonstrated that ectopic expression of Ven-HROB or HROB-Ven demonstrated a rapid reduction in EdU-positive (i.e., S phase) cells, whereas the fraction of S-phase cells among the HROB-negative cells in the same culture or in cells expressing Ven alone was unchanged (Figure 5C,D). This observed reduced proportion of EdU^+^ in the HROB-expressing cells is reflected in the cell cycle analysis in a corresponding reduction in S phase cells in these samples (Figure 5E). Interestingly, neither a clear accumulation of G1 nor G2 phase cells could be observed in the HROB-expressing cells, arguing against a specific G1 or G2 arrest of the cell cycle (Figure 5C–E). Considering the unusual phenotype, the effect of overexpression of HROB was confirmed independently in U2OS cells, indicating that the effect is not cell-type-specific (Supplementary Figure S14).

**Figure 6:**
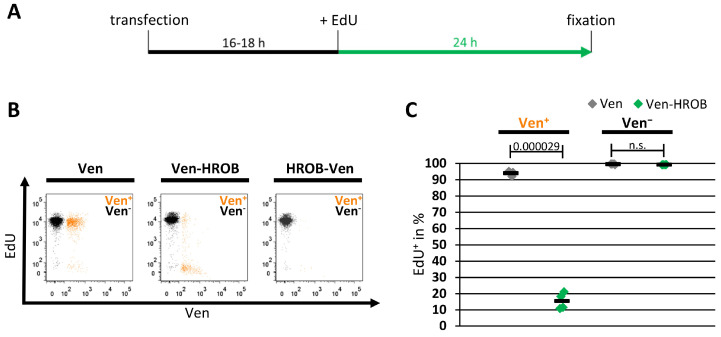
HROB-overexpressing cells show a dramatic reduction in DNA replication. HEp-2 cells were transiently transfected with plasmids encoding Venus (Ven), Ven-HROB or HROB-Ven. An amount of 16–18 h after transfection, the cells were treated with 10 μM EdU for 24 h and then analyzed by flow cytometry after EdU and DAPI staining (n = 4). (**A**) Representation of the experimental workflow. (**B**) The intensities determined for EdU staining and for Ven are plotted against each other. Ven-positive (Ven^+^) cells are shown in orange, Ven-negative (Ven^−^) cells in black (representative diagrams of one of four independent biological samples). (**C**) For each sample, Ven^+^ and Ven^−^ were distinguished and analyzed for the proportion of EdU-positive cells (EdU^+^). Each data point represents an independent biological sample. The means are represented by black bars. The *p* values were determined using two-sided Student’s *t*-tests (n.s. – not significant).

To determine whether the observed reduced proportion of EdU^+^ cells in HROB-overexpressing cells was indeed due to a reduced initiation of DNA synthesis, the transfected cells were subjected to long-term EdU treatment (Figure 6A). For this purpose, the cells were treated 16–18 h after transfection with EdU for 24 h to mark all cells that have shown DNA synthesis activity during this period. As expected, a vast majority of control cells expressing Ven alone initiated DNA replication. In contrast, the Ven^+^ population of the cells transfected to express Ven-HROB showed a significantly lower proportion of EdU-positive averaging 15%. At the same time, the proportion of EdU^+^ cells was not reduced in the Ven-negative cells of same samples (Figure 6B,C). Overall, these results demonstrated that DNA replication comes to an almost complete standstill in cells with an increased protein level of HROB.

## 4. Discussion

In this study, we focused on HROB, which we identified as a protein with a common expression regulation with many factors of DNA replication and repair. In accordance, we characterized HROB as a nuclear, chromatin-bound protein showing an S-phase-specific and proliferation-associated expression with downregulation in quiescent cells and upregulation in many different cancer entities. HROB is well conserved within eukaryotes, and structural prediction and homology searches revealed a putative OB-fold motif extended by a preceding helical region within the otherwise largely intrinsically disordered protein. The detected FRET association of HROB with proteins of the elongating replication fork and the finding that a strongly elevated cellular HROB level leads to rapid and dramatic reduction in replicative DNA synthesis indicate a function of HROB connected with DNA replication.

Recent reports of several groups identified HROB as a binding partner and activator of the Mcm8/9 helicase involved in the DNA repair of ICL [36,48,98]. HROB knock-out mice were infertile due to meiotic arrests and subsequent depletion of germ cells [36], and the loss of HROB confers cellular sensitivity to cisplatin, mitomycin C, psoralene plus UV-A, etoposide and olaparib [31,36,48]. Whereas no changes in cell cycle progression or growth rate were detected in our HROB knock-down model (Supplementary Figure S13), the cellular knock-out of HROB was associated with reduced cell growth, decreased BrdU incorporation and replication progression and increased H2AX phosphorylation under untreated conditions [36,48]. The treatment of HROB knock-out cells with cisplatin or mitomycin C led to elevated levels of DNA damage signaling, increased fork stalling, aberrant mitosis with anaphase bridges and lagging chromosomes and G2/M accumulation. These effects were accompanied by the formation of more and longer-persisting foci of γH2AX, Rad51 and FANCD2 compared to WT cells, suggesting a function of HROB in HR [31,36,48].

On the other hand, HROB does not behave like a classical HR factor, since the knock-out cells are not sensitized for irradiation, camptothecin or hydroxyurea [36,48,98]. Instead, they exclusively show increased sensitivity to DNA-damaging agents that insert fork-blocking lesions, considering that etoposide is hypothesized to form replication-fork-blocking obstacles by trapping topoisomerase on DNA [99,100,101] and that olaparib is known to trap PARP1 at damage sites [102,103]. This suggests a function for HROB in handling fork barriers during the process of DNA replication.

In concordance, HROB is a proliferation-related protein robustly co-regulated with proteins of DNA replication and replication-associated repair (Supplementary Figure S1), and we detected a strong reduction in the HROB protein level in quiescent cells compared to proliferative cells (Figure 3D,E), which are both consistent with a previous report [48]. In contradiction to this report, in which no changes in the cellular protein level of HROB were observed in thymidine-synchronized 293A cells [48], we detected an elevated HROB protein level within the S phase in HEp-2 cells synchronized by G2 arrests utilizing a Cdk1 inhibitor, and a strong correlation between the protein levels of HROB and Cyclin A, indicating an S-phase-specific expression of HROB (Figure 3A–C).

ICLs are potent fork barriers, and collision of CMG with the lesion activates the Fanconi anemia pathway [24,104]. The collision can either directly trigger the repair, whereby a one-ended DSB is formed, and CMG is released via the DNA incision on both sides of the ICL. Subsequently, a translesion polymerase bypasses the monoadduct generated by unhooking of the ICL; the monoadduct is removed by nucleotide excision repair, and HR restores the replication fork [104,105]. Alternatively, ICL repair may require the convergence of two replication forks on the ICL, followed by CMG ubiquitinylation and unloading, where a two-ended DSB is generated by the DNA incision upstream and downstream of the ICL. The subsequent repair of the unhooked ICL also involves translesion synthesis and HR and may additionally require nucleotide excision repair [24,104].

However, the CMG helicase can also ‘traverse’ an ICL, and DNA-combing assays with simultaneous visualization of ICL demonstrated that in 50–70% of the observed ICL structures, the replication fork had passed through the intact ICL [104,106,107]. The molecular details that take place during the traversal of ICLs are just starting to be uncovered. ICL traversal also depends on ATR and the Fanconi anemia pathway and involves, among other proteins, FANCM, FANCD2 and PRIMPOL [108,109]. CMG remodeling may facilitate the opening of the Mcm2-7 ring so it can translocate the ICL if another DNA helicase generates ssDNA adjacent to the lesion [24,104].

Given the requirement for an additional helicase in ICL traversing, the HROB-Mcm8/9 complex could be involved in installing the replication fork beyond the ICL [24,31]. This is supported by the facts that Mcm8/9 primarily confers resistance to cisplatin and mitomycin C and that its loss is associated with a reduction in growth rate, a slowdown of DNA replication and an increase in collapsing and stalling of replication forks [30,37,45,47]. The HROB-Mcm8/9 complex displays a distinct binding and unwinding preference for branched DNA structures [49]. Mcm8/9 enables residual replicative DNA synthesis in a system where Mcm2 is conditionally degraded by an auxin–degron approach and depletion of HROB abrogated that residual synthesis [36,38]. Moreover, Mcm8 and 9 were both shown to be significantly enriched on active replisomes [110] and co-immunoprecipitated with RFC [111]. A loss of PRIMPOL during ICL traversal is associated with the formation of more and longer-persisting FANCD2 foci, such as was observed for cells lacking HROB [48,108]. Furthermore, the mitomycin C-induced formation of Mcm9 foci was strongly diminished in cells lacking FANCD2 [112].

The observations that the depletion of HROB abolishes the recruitment of Mcm8 to DNA repair sites and that HROB increases the affinity of the Mcm8/9 helicase for ssDNA suggest that HROB facilitates the loading of Mcm8/9 at DNA damage sites [31,36]. Inline, HROB shows a strong chromatin association (Figure 1A,B) despite a weak DNA-binding activity by itself in vitro [31,36,48]. Thus, chromatin binding appears to be mediated by other proteins. Here, we could detect the FRET association of HROB to the DNA replication proteins Cdc45, RPA32, Mcm5 and Mcm2, indicating an association of HROB with the replisome (Figure 4). The interaction between HROB and RPA was validated in several reports via pull-down and co-immunoprecipitation experiments [31,36,48,113]. Furthermore, the FRET association to Mcm5 was also confirmed by others as a significant enrichment of this protein in a HROB-Co-IP-MS analysis, where additionally an association to the replisome proteins Mcm7, RFC1/3/5 and PolDIP2/3 was detected [31]. The fact that an association of HROB with Cdc45 was not detected in the Co-IP performed there could be explained by the circumstance that Cdc45, as limiting factor of DNA replication, is only present in a low molecular number per cell [86,95,96] and can therefore also be poorly recorded by mass spectrometry in whole cell lysates. Thus, even in CoIP-MS experiments with Mcm2 or Mcm5, no enrichment of Cdc45 was detected [114,115]. The observed interaction with multiple replication factors could explain why an HROB mutant that cannot bind RPA still remained associated with chromatin despite its very weak intrinsic DNA affinity [31].

HROB also provides the structural requirements for the interaction with many different binding partners. HROB is predicted to be a largely disordered protein, as indicated by the DISOPRED analyses performed here (Figure 1C). As IDR are characterized by low content of non-polar and many-charged amino acids, predictions of IDR made from the protein sequence have been shown to be accurate [116,117]. The presence of IDRs within HROB is conserved in all studied vertebrate homologs, stretching the importance of these protein segments for the HROB function (Figure 1D). IDR-containing proteins have special properties due to their extreme flexibility and structural instability, which have a particular impact on their interaction possibilities. IDRs allow highly specific binding with fast association rates to a large number of different binding partners with various structures. That is why intrinsically disordered proteins are often involved in the formation of complex protein networks or molecular condensates and fulfill regulatory functions [118,119,120].

Based on structural predictions and interaction studies with mutants, individual protein segments of HROB could be assigned to individual interactions in previous reports. According to AlphaFold predictions, the OB-fold could mediate the binding to Mcm9 [49] and a mutant lacking R396–S413 suggested that the interaction to Mcm8/9 is further mediated by the helix-rich region preceding the OB-fold [31]. As shown by the creation of a non-RPA-binding mutant, the acidic patches within the HROB N-terminal part enable its interaction to RPA [31].

Even when HROB does not seem to possess the structural and enzymatic properties for helicase loading and does not show sequence similarities to helicase-loading factors such as DnaA or ORC [121,122], the interaction of HROB to Mcm8/9 could support the association of the helicase and regulate its activity. Thereby, the high flexibility of the HROB structure may allow the interaction to Mcm8/9, to RPA or to other replisome components in parallel.

**Figure 7:**
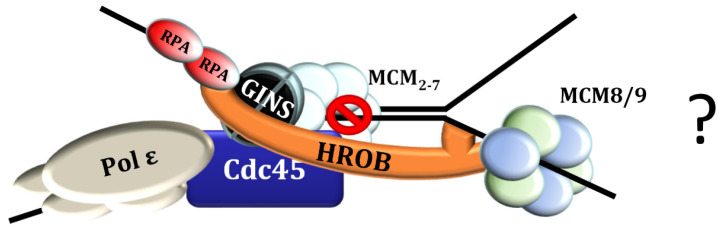
A model of HROB facilitating the association of Mcm8/9 and locally restricting its activity by simultaneous interaction to the helicase and to the replisome during ICL traversing. This model is based on the observations and conclusions listed as follows: (i) A major fraction of ICL replication conflicts are resolved via ICL traversal, where the fork passes through the intact ICL, and DNA replication is continued beyond the lesion. This requires the remodeling of the CMG helicase and the reconstruction of the replication fork beyond the ICL. (ii) To prevent over-replication, the cell must ensure that new replication forks are exclusively formed where other forks have been stalled. The establishment of the new replication fork requires the unwinding of the DNA behind/beyond the ICL. Mcm8/9 is a helicase whose knock-out sensitizes cells to ICL, and it is enriched at replisomes and localizes at ICL damage sites. HROB is the Mcm8/9 activator whose knock-out also sensitizes cells to ICL. HROB associates with DNA replication factors and localizes at ICL damage sites. The depletion of HROB abolishes the recruitment of Mcm8 to DNA repair sites. (iii) HROB is an intrinsically disordered protein, and its high structural flexibility may allow the simultaneous interaction to multiple different binding partners. (iv) ICL traversal is dependent on FANCD2. The depletion of FANCD2 diminishes the association of HROB and of Mcm9 with ICL damage sites and the knock-out of HROB leads to the persistence of FANCD2 foci. The question mark on the right-hand side indicate that there are uncertainties in the model that require further research. See text for details and references.

Since HROB functions as an activator of the Mcm8/9 helicase and enhances not only its affinity for ssDNA but also its helicase activity [31,49], a possible model is that HROB supports the Mcm8/9 recruitment to replication barriers and, at the same time, guarantees that the activation of Mcm8/9 is limited to stalled forks via its association with the replisome (Figure 7). This model could also explain the inhibitory effect of HROB overexpression on DNA replication, since increased levels may disturb the regulation of the HROB-Mcm8/9 complex leading to excessive loading of the back-up helicase at undesired sites (see also below). In order to maintain genetic integrity, it is essential to ensure that the installation of new replication forks in the context of the ICL traversing is tightly restricted to the locations of “failed” forks at blocked replication sites to prevent over-replication.

The proposed model does not explain why depletion of HROB was also associated with a reduction in the HR capacity after *Sce*I-induced DSB [31,48], nor why the localization of HROB on DNA damage sites was reduced after knock-down of CtIP [31,36]. This indicates that HROB may also stimulate Mcm8/9 in different contexts of DNA repair, and that an ICL-traversal-related mechanism may be employed in different situations where the establishment of an alternative replication fork may be required, also providing an alternative to break-induced replication [123].

The results of the cellular overexpression of HROB also support a function of HROB in the context of DNA replication. The effect on DNA replication was observed equally using both N- and C-terminally tagged HROB, making a negative influence of the fusion extremely unlikely. The proportion of HROB-positive cells was significantly reduced in a time-dependent manner compared to controls, and at the same time, neither cell cycle arrest nor increase in cleaved PARP nor enhanced γH2AX was observed, leading to the assumption that HROB-overexpressing cells exhibit reduced growth (Figure 5). In consistence, long-term monitoring of DNA synthesis activity showed that DNA replication comes to an almost complete shutdown when the HROB protein level is elevated (Figure 6). The dramatic effect of the HROB-overexpression is unexpected because transient overexpression of proteins is permissible. It is a way reminiscent of the toxic effect of increased levels of the critical replication and HR factors Cdc45 [86,124], RPA [125] and Rad51 [97,126]. In contrast, elevated levels of several other replication and HR proteins such as Orc2 [127], Mcm2/5/10 [94,128,129], GINS [130], PCNA [131], RecQL4 [132], TopBP1 [133], or FANCA/C/D2 [134,135,136,137] are well tolerated. The observed phenotype is different from the effect of downregulation of RPA or Cdc45 [138,139], Therefore, it is unlikely that HROB overexpression just sequesters Cdc45 or RPA. Taken together, these results suggest that DNA replication is not disturbed but rather not initiated after HROB overexpression.

In summary, we propose, based on our observations in combination with previously reported results, a role for HROB together with Mcm8/9 in handling fork barriers during DNA replication by establishing an alternative helicase behind the obstacle. The observed associations of HROB with multiple DNA replication proteins and the impressively strong decrease in DNA replication upon HROB overexpression indicate that the function of HROB is closely linked to the elongation of DNA replication.

## Data Availability

The original contributions presented in the study are included in the article/Supplementary Material; further inquiries can be directed to the corresponding author. All plasmids generated in this study have been made publicly available via Addgene.

## Supplementary Materials

The following supporting information can be downloaded at: https://www.mdpi.com/article/10.3390/genes15121587/s1: Figure S1: HROB shares expression regulation with several DNA replication proteins; Figure S2: Illustration of the secondary structure prediction of HROB; Figure S3: The structure of HROB is conserved in eukaryotes; Figure S4: Protein fragments identified by the random PCR screen are consistent with the HROB structure prediction; Figure S5: The HROB protein level is upregulated in cancer and proliferating cells; Figure S6: Influence of the expression level of HROB on the probability of survival in different cancers; Figure S7: Verification of the correct expression of the generated fluorescent fusion proteins; Figure S8: Verification of the nuclear localization of the generated fluorescent fusion proteins; Figure S9: The principle of the flow cytometry FRET assay; Figure S10: Utilized gating schemes for the analysis of flow cytometric experiments; Figure S11: The flow cytometry FRET assay; Figure S12: Estimation of the number of HROB molecules per cell; Figure S13: Knock-down of HROB does not affect cell cycle distribution or growth rate; Figure S14: HROB-overexpressing U2OS cells show an S phase depletion; Table S1: List of primers used for the generation of plasmids; Table S2: Protein-ID of HROB homologs shown in Figure 1; Table S3: Protein-ID of HROB homologs shown in Supplementary Figure S3; Table S4: Quantification of the FRET experiments presented in Figure 4 and Supplementary Figure S11; Table S5: Overview of the values determined for single cells (cells), Cerulean- and Venus-double-positive cells (Cer^+^ and Ven^+^) and FRET^+^ cells (FRET^+^) in the cytometry FRET assays with HROB fusion proteins (corresponding to Figure 4); Table S6: Overview of the values determined for single cells (cells), Cerulean- and Venus-double-positive cells (Cer^+^ and Ven^+^) and FRET^+^ cells (FRET^+^) in the cytometry FRET assays with Cdc45 fusion proteins (corresponding to Supplementary Figure S11). Refs. [140,141,142] are cited in Supplementary Materials.

## References

[1] Abbas T., Keaton M.A., Dutta A. (2013). Genomic Instability in Cancer. Cold Spring Harb. Perspect. Biol..

[2] Gaillard H., García-Muse T., Aguilera A. (2015). Replication Stress and Cancer. Nat. Rev. Cancer.

[3] Saxena S., Zou L. (2022). Hallmarks of DNA Replication Stress. Mol. Cell.

[4] Fragkos M., Ganier O., Coulombe P., Méchali M. (2015). DNA Replication Origin Activation in Space and Time. Nat. Rev. Mol. Cell Biol..

[5] Lin Y.C., Prasanth S.G. (2021). Replication Initiation: Implications in Genome Integrity. DNA Repair.

[6] Parker M.W., Botchan M.R., Berger J.M. (2017). Mechanisms and Regulation of DNA Replication Initiation in Eukaryotes. Crit. Rev. Biochem. Mol. Biol..

[7] Kang S., Kang M.S., Ryu E., Myung K. (2018). Eukaryotic DNA Replication: Orchestrated Action of Multi-Subunit Protein Complexes. Mutat. Res..

[8] Ishimi Y. (2018). Regulation of MCM2-7 Function. Genes Genet. Syst..

[9] Ilves I., Petojevic T., Pesavento J.J., Botchan M.R. (2010). Activation of the MCM2-7 Helicase by Association with Cdc45 and GINS Proteins. Mol. Cell.

[10] Costa A., Ilves I., Tamberg N., Petojevic T., Nogales E., Botchan M.R., Berger J.M. (2011). The Structural Basis for MCM2-7 Helicase Activation by GINS and Cdc45. Nat. Struct. Mol. Biol..

[11] Gambus A., Jones R.C., Sanchez-Diaz A., Kanemaki M., van Deursen F., Edmondson R.D., Labib K. (2006). GINS Maintains Association of Cdc45 with MCM in Replisome Progression Complexes at Eukaryotic DNA Replication Forks. Nat. Cell Biol..

[12] Moyer S.E., Lewis P.W., Botchan M.R. (2006). Isolation of the Cdc45/Mcm2-7/GINS (CMG) Complex, a Candidate for the Eukaryotic DNA Replication Fork Helicase. Proc. Natl. Acad. Sci. USA.

[13] Tanaka S., Umemori T., Hirai K., Muramatsu S., Kamimura Y., Araki H. (2007). CDK-Dependent Phosphorylation of Sld2 and Sld3 Initiates DNA Replication in Budding Yeast. Nature.

[14] Kumagai A., Shevchenko A., Shevchenko A., Dunphy W.G. (2011). Direct Regulation of Treslin by Cyclin-Dependent Kinase Is Essential for the Onset of DNA Replication. J. Cell Biol..

[15] Francis L.I., Randell J.C.W., Takara T.J., Uchima L., Bell S.P. (2009). Incorporation into the Prereplicative Complex Activates the Mcm2-7 Helicase for Cdc7-Dbf4 Phosphorylation. Genes Dev..

[16] Masai H., Taniyama C., Ogino K., Matsui E., Kakusho N., Matsumoto S., Kim J.M., Ishii A., Tanaka T., Kobayashi T. (2006). Phosphorylation of MCM4 by Cdc7 Kinase Facilitates Its Interaction with Cdc45 on the Chromatin. J. Biol. Chem..

[17] Besnard E., Babled A., Lapasset L., Milhavet O., Parrinello H., Dantec C., Marin J.-M., Lemaitre J.-M. (2012). Unraveling Cell Type-Specific and Reprogrammable Human Replication Origin Signatures Associated with G-Quadruplex Consensus Motifs. Nat. Struct. Mol. Biol..

[18] Langley A.R., Gräf S., Smith J.C., Krude T. (2016). Genome-Wide Identification and Characterisation of Human DNA Replication Origins by Initiation Site Sequencing (Ini-Seq). Nucleic Acids Res..

[19] Chagin V.O., Casas-Delucchi C.S., Reinhart M., Schermelleh L., Markaki Y., Maiser A., Bolius J.J., Bensimon A., Fillies M., Domaing P. (2016). 4D Visualization of Replication Foci in Mammalian Cells Corresponding to Individual Replicons. Nat. Commun..

[20] Méchali M. (2010). Eukaryotic DNA Replication Origins: Many Choices for Appropriate Answers. Nat. Rev. Mol. Cell Biol..

[21] Moiseeva T.N., Bakkenist C.J. (2019). Dormant Origin Signaling during Unperturbed Replication. DNA Repair.

[22] Marchal C., Sima J., Gilbert D.M. (2019). Control of DNA Replication Timing in the 3D Genome. Nat. Rev. Mol. Cell Biol..

[23] Chakraborty S., Schirmeisen K., Lambert S.A. (2023). The Multifaceted Functions of Homologous Recombination in Dealing with Replication-Associated DNA Damages. DNA Repair.

[24] Cortez D. (2019). Replication-Coupled DNA Repair. Mol. Cell.

[25] Spies J., Polasek-Sedlackova H., Lukas J., Somyajit K. (2021). Homologous Recombination as a Fundamental Genome Surveillance Mechanism during DNA Replication. Genes.

[26] Griffin W.C., Trakselis M.A. (2019). The MCM8/9 Complex: A Recent Recruit to the Roster of Helicases Involved in Genome Maintenance. DNA Repair.

[27] Li Z., Xu X. (2019). Post-Translational Modifications of the Mini-Chromosome Maintenance Proteins in DNA Replication. Genes.

[28] Nishimura K., Ishiai M., Horikawa K., Fukagawa T., Takata M., Takisawa H., Kanemaki M.T. (2012). Mcm8 and Mcm9 Form a Complex That Functions in Homologous Recombination Repair Induced by DNA Interstrand Crosslinks. Mol. Cell.

[29] Weng Z., Zheng J., Zhou Y., Lu Z., Wu Y., Xu D., Li H., Liang H., Liu Y. (2023). Structural and Mechanistic Insights into the MCM8/9 Helicase Complex. eLife.

[30] Helderman N.C., Terlouw D., Bonjoch L., Golubicki M., Antelo M., Morreau H., van Wezel T., Castellví-Bel S., Goldberg Y., Nielsen M. (2023). Molecular Functions of MCM8 and MCM9 and Their Associated Pathologies. iScience.

[31] Huang J.W., Acharya A., Taglialatela A., Nambiar T.S., Cuella-Martin R., Leuzzi G., Hayward S.B., Joseph S.A., Brunette G.J., Anand R. (2020). MCM8IP Activates the MCM8-9 Helicase to Promote DNA Synthesis and Homologous Recombination upon DNA Damage. Nat. Commun..

[32] Kinoshita Y., Johnson E.M., Gordon R.E., Negri-bell H., Evans M.T., Coolbaugh J., Rosario-peralta Y., Samet J., Slusser E., Birkenbach M.P. (2007). Colocalization of MCM8 and MCM7 With Proteins Involved in Distinct Aspects of DNA Replication. Microsc. Res. Tech..

[33] Lutzmann M., Méchali M. (2008). MCM9 Binds Cdt1 and Is Required for the Assembly of Prereplication Complexes. Mol. Cell.

[34] Volkening M., Hoffmann I. (2005). Involvement of Human MCM8 in Prereplication Complex Assembly by Recruiting Hcdc6 to Chromatin. Mol. Cell. Biol..

[35] Maiorano D., Cuvier O., Danis E., Méchali M. (2005). MCM8 Is an MCM2-7-Related Protein That Functions as a DNA Helicase during Replication Elongation and Not Initiation. Cell.

[36] Hustedt N., Saito Y., Zimmermann M., Álvarez-Quilón A., Setiaputra D., Adam S., McEwan A., Yuan J.Y., Olivieri M., Zhao Y. (2019). Control of Homologous Recombination by the HROB–MCM8–MCM9 Pathway. Genes Dev..

[37] Lutzmann M., Grey C., Traver S., Ganier O., Maya-Mendoza A., Ranisavljevic N., Bernex F., Nishiyama A., Montel N., Gavois E. (2012). MCM8- and MCM9-Deficient Mice Reveal Gametogenesis Defects and Genome Instability Due to Impaired Homologous Recombination. Mol. Cell.

[38] Natsume T., Nishimura K., Minocherhomji S., Bhowmick R., Hickson I.D., Kanemaki M.T. (2017). Acute Inactivation of the Replicative Helicase in Human Cells Triggers MCM8-9-Dependent DNA Synthesis. Genes Dev..

[39] Lee K.Y., Im J.S., Shibata E., Park J., Handa N., Kowalczykowski S.C., Dutta A. (2015). MCM8-9 Complex Promotes Resection of Double-Strand Break Ends by MRE11-RAD50-NBS1 Complex. Nat. Commun..

[40] Park J., Long D.T., Lee K.Y., Abbas T., Shibata E., Negishi M., Luo Y., Schimenti J.C., Gambus A., Walter J.C. (2013). The MCM8-MCM9 Complex Promotes RAD51 Recruitment at DNA Damage Sites to Facilitate Homologous Recombination. Mol. Cell. Biol..

[41] McKinzey D.R., Gomathinayagam S., Griffin W.C., Klinzing K.N., Jeffries E.P., Rajkovic A., Trakselis M.A. (2021). Motifs of the C-terminal Domain of MCM9 Direct Localization to Sites of Mitomycin-C Damage for RAD51 Recruitment. J. Biol. Chem..

[42] Morii I., Iwabuchi Y., Mori S., Suekuni M., Natsume T., Yoshida K., Sugimoto N., Kanemaki M.T., Fujita M. (2019). Inhibiting the MCM8-9 Complex Selectively Sensitizes Cancer Cells to Cisplatin and Olaparib. Cancer Sci..

[43] Desai S., Wood-Trageser M., Matic J., Chipkin J., Jiang H., Bachelot A., Dulon J., Sala C., Barbieri C., Cocca M. (2017). MCM8 and MCM9 Nucleotide Variants in Women With Primary Ovarian Insufficiency. J. Clin. Endocrinol. Metab..

[44] Hartford S.A., Luo Y., Southard T.L., Min I.M., Lis J.T., Schimenti J.C. (2011). Minichromosome Maintenance Helicase Paralog MCM9 Is Dispensible for DNA Replication but Functions in Germ-Line Stem Cells and Tumor Suppression. Proc. Natl. Acad. Sci. USA.

[45] Lutzmann M., Bernex F., da Costa de Jesus C., Hodroj D., Marty C., Plo I., Vainchenker W., Tosolini M., Forichon L., Bret C. (2019). MCM8- and MCM9 Deficiencies Cause Lifelong Increased Hematopoietic DNA Damage Driving P53-Dependent Myeloid Tumors. Cell Rep..

[46] Gambus A., Blow J. (2013). Mcm8 and Mcm9 Form a Dimeric Complex in Xenopus Laevis Egg Extract That Is Not Essential for DNA Replication Initiation. Cell Cycle.

[47] Griffin W.C., McKinzey D.R., Klinzing K.N., Baratam R., Eliyapura A., Trakselis M.A. (2022). A Multi-Functional Role for the MCM8/9 Helicase Complex in Maintaining Fork Integrity during Replication Stress. Nat. Commun..

[48] Wang C., Chen Z., Su D., Tang M., Nie L., Zhang H., Feng X., Wang R., Shen X., Srivastava M. (2020). C17orf53 Is Identified as a Novel Gene Involved in Inter-Strand Crosslink Repair. DNA Repair.

[49] Acharya A., Bret H., Huang J.W., Mütze M., Göse M., Kissling V.M., Seidel R., Ciccia A., Guérois R., Cejka P. (2024). Mechanism of DNA Unwinding by MCM8-9 in Complex with HROB. Nat. Commun..

[50] Koushik S.V., Chen H., Thaler C., Puhl H.L., Vogel S.S. (2006). Cerulean, Venus, and VenusY67C FRET Reference Standards. Biophys. J..

[51] Keller H., Kiosze K., Sachsenweger J., Haumann S., Ohlenschläger O., Nuutinen T., Syväoja J.E., Görlach M., Grosse F., Pospiech H. (2014). The Intrinsically Disordered Amino-Terminal Region of Human RecQL4: Multiple DNA-Binding Domains Confer Annealing, Strand Exchange and G4 DNA Binding. Nucleic Acids Res..

[52] Altschul S.F., Gish W., Miller W., Myers E.W., Lipman D.J. (1990). Basic Local Alignment Search Tool. J. Mol. Biol..

[53] Hassinen A., Pujol F.M., Kokkonen N., Pieters C., Kihlström M., Korhonen K., Kellokumpu S. (2011). Functional Organization of Golgi N- and O-Glycosylation Pathways Involves PH-Dependent Complex Formation That Is Impaired in Cancer Cells. J. Biol. Chem..

[54] Van Wageningen S., Pennings A.H., Van Der Reijden B.A., Boezeman J.B., De Lange F., Jansen J.H. (2006). Isolation of FRET-Positive Cells Using Single 408-Nm Laser Flow Cytometry. Cytom. Part A.

[55] Sarkar P., Koushik S.V., Vogel S.S., Gryczynski I., Gryczynski Z. (2009). Photophysical Properties of Cerulean and Venus Fluorescent Proteins. J. Biomed. Opt..

[56] Jones D.T., Cozzetto D. (2015). DISOPRED3: Precise Disordered Region Predictions with Annotated Protein-Binding Activity. Bioinformatics.

[57] Jones D.T. (1999). Protein Secondary Structure Prediction Based on Position-Specific Scoring Matrices. J. Mol. Biol..

[58] Potter S.C., Luciani A., Eddy S.R., Park Y., Lopez R., Finn R.D. (2018). HMMER Web Server: 2018 Update. Nucleic Acids Res..

[59] Madeira F., Park Y.M., Lee J., Buso N., Gur T., Madhusoodanan N., Basutkar P., Tivey A.R.N., Potter S.C., Finn R.D. (2019). The EMBL-EBI Search and Sequence Analysis Tools APIs in 2019. Nucleic Acids Res..

[60] Altschul S.F., Madden T.L., Schäffer A.A., Zhang J., Zhang Z., Miller W., Lipman D.J. (1997). Gapped BLAST and PSI-BLAST: A New Generation of Protein Database Search Programs. Nucleic Acids Res..

[61] Zerbino D.R., Achuthan P., Akanni W., Amode M.R., Barrell D., Bhai J., Billis K., Cummins C., Gall A., Girón C.G. (2018). Ensembl 2018. Nucleic Acids Res..

[62] El-Gebali S., Mistry J., Bateman A., Eddy S.R., Luciani A., Potter S.C., Qureshi M., Richardson L.J., Salazar G.A., Smart A. (2019). The Pfam Protein Families Database in 2019. Nucleic Acids Res..

[63] Sayers E.W., Bolton E.E., Brister J.R., Canese K., Chan J., Comeau D.C., Farrell C.M., Feldgarden M., Fine A.M., Funk K. (2023). Database Resources of the National Center for Biotechnology Information in 2023. Nucleic Acids Res..

[64] Hinchliff C.E., Smith S.A., Allman J.F., Burleigh J.G., Chaudhary R., Coghill L.M., Crandall K.A., Deng J., Drew B.T., Gazis R. (2015). Synthesis of Phylogeny and Taxonomy into a Comprehensive Tree of Life. Proc. Natl. Acad. Sci. USA.

[65] Sotero-Caio C.G., Platt R.N., Suh A., Ray D.A. (2017). Evolution and Diversity of Transposable Elements in Vertebrate Genomes. Genome Biol. Evol..

[66] Méndez J., Stillman B. (2000). Chromatin Association of Human Origin Recognition Complex, Cdc6, and Minichromosome Maintenance Proteins during the Cell Cycle: Assembly of Prereplication Complexes in Late Mitosis. Mol. Cell. Biol..

[67] Bauerschmidt C., Pollok S., Kremmer E., Nasheuer H.-P., Grosse F. (2007). Interactions of Human Cdc45 with the Mcm2–7 Complex, the GINS Complex, and DNA Polymerases δ and ε during S Phase. Genes Cells.

[68] Ohlenschläger O., Kuhnert A., Schneider A., Haumann S., Bellstedt P., Keller H., Saluz H.-P., Hortschansky P., Hänel F., Grosse F. (2012). The N-Terminus of the Human RecQL4 Helicase Is a Homeodomain-like DNA Interaction Motif. Nucleic Acids Res.

[69] Kenny M.K., Schlegel U., Furneaux H., Hurwitz J. (1990). The Role of Human Single-Stranded DNA Binding Protein and Its Individual Subunits in Simian Virus 40 DNA Replication. J. Biol. Chem..

[70] Mäkiniemi M., Hillukkala T., Tuusa J., Reini K., Vaara M., Huang D., Pospiech H., Majuri I., Westerling T., Mäkelä T.P.P. (2001). BRCT Domain-Containing Protein TopBP1 Functions in DNA Replication and Damage Response. J. Biol. Chem..

[71] Pers T.H., Karjalainen J.M., Chan Y., Westra H.J., Wood A.R., Yang J., Lui J.C., Vedantam S., Gustafsson S., Esko T. (2015). Biological Interpretation of Genome-Wide Association Studies Using Predicted Gene Functions. Nat. Commun..

[72] Lou J., Chen H., Han J., He H., Huen M.S.Y., Feng X.H., Liu T., Huang J. (2017). AUNIP/C1orf135 Directs DNA Double-Strand Breaks towards the Homologous Recombination Repair Pathway. Nat. Commun..

[73] Tran P.T., Fey J.P., Erdeniz N., Gellon L., Boiteux S., Liskay R.M. (2007). A Mutation in EXO1 Defines Separable Roles in DNA Mismatch Repair and Post-Replication Repair. DNA Repair.

[74] Laoukili J., Stahl M., Medema R.H. (2007). FoxM1: At the Crossroads of Ageing and Cancer. Biochim. Biophys. Acta.

[75] Musa J., Aynaud M.M., Mirabeau O., Delattre O., Grünewald T.G.P. (2017). MYBL2 (B-Myb): A Central Regulator of Cell Proliferation, Cell Survival and Differentiation Involved in Tumorigenesis. Cell Death Dis..

[76] Yang S., Liu X., Yin Y., Fukuda M.N., Zhou J. (2008). Tastin Is Required for Bipolar Spindle Assembly and Centrosome Integrity during Mitosis. FASEB J..

[77] Colicino E.G., Hehnly H. (2018). Regulating a Key Mitotic Regulator, Polo-like Kinase 1 (PLK1). Cytoskeleton.

[78] Bass D., Czech L., Williams B.A.P., Berney C., Dunthorn M., Mahé F., Torruella G., Stentiford G.D., Williams T.A. (2018). Clarifying the Relationships between Microsporidia and Cryptomycota. J. Eukaryot. Microbiol..

[79] Baldauf S.L. (2003). The Deep Roots of Eukaryotes. Science.

[80] Karlsson M., Zhang C., Méar L., Zhong W., Digre A., Katona B., Sjöstedt E., Butler L., Odeberg J., Dusart P. (2021). A Single–Cell Type Transcriptomics Map of Human Tissues. Sci. Adv..

[81] Clegg R.M. (1995). Fluorescence Resonance Energy Transfer. Curr. Opin. Biotechnol..

[82] Piehler J. (2005). New Methodologies for Measuring Protein Interactions in Vivo and in Vitro. Curr. Opin. Struct. Biol..

[83] Shrestha D., Jenei A., Nagy P., Vereb G., Szöllősi J. (2015). Understanding FRET as a Research Tool for Cellular Studies. Int. J. Mol. Sci..

[84] Piergiovanni G., Costanzo V., Maurer-Stroh S. (2010). GEMC1 Is a Novel TopBP1-Interacting Protein Involved in Chromosomal DNA Replication. Cell Cycle.

[85] Champeris Tsaniras S., Kanellakis N., Symeonidou I.E., Nikolopoulou P., Lygerou Z., Taraviras S. (2014). Licensing of DNA Replication, Cancer, Pluripotency and Differentiation: An Interlinked World?. Semin. Cell Dev. Biol..

[86] Köhler C., Koalick D., Fabricius A., Parplys A.C., Borgmann K., Pospiech H., Grosse F. (2016). Cdc45 Is Limiting for Replication Initiation in Humans. Cell Cycle.

[87] Mantiero D., Mackenzie A., Donaldson A., Zegerman P. (2011). Limiting Replication Initiation Factors Execute the Temporal Programme of Origin Firing in Budding Yeast. EMBO J..

[88] Liu K., Graves J.D., Lee Y.-J., Lin F.-T., Lin W.-C. (2020). Cell Cycle-Dependent Switch of TopBP1 Functions by Cdk2 and Akt. Mol. Cell. Biol..

[89] Mughal M.J., Mahadevappa R., Kwok H.F. (2019). DNA Replication Licensing Proteins: Saints and Sinners in Cancer. Semin. Cancer Biol..

[90] Burkhart R., Schulte D., Hu B., Musahl C., Göhring F., Knippers R. (1995). Interactions of Human Nuclear Proteins P1Mcm3 and P1Cdc46. Eur. J. Biochem..

[91] Seroussi E., Laviz S. (1993). Replication Protein A Is the Major Single-Stranded DNA Binding Protein Detected in Mammalian Cell Extracts by Gel Retardation Assays and UV Cross-Linking of Long and Short Single-Stranded DNA Molecules. J. Biol. Chem..

[92] Wong P.G., Winter S.L., Zaika E., Cao T.V., Oguz U., Koomen J.M., Hamlin J.L., Alexandrow M.G. (2011). Cdc45 Limits Replicon Usage from a Low Density of PreRCs in Mammalian Cells. PLoS ONE.

[93] Xouri G., Lygerou Z., Nishitani H., Pachnis V., Nurse P., Taraviras S. (2004). Cdt1 and Geminin Are Down-Regulated upon Cell Cycle Exit and Are over-Expressed in Cancer-Derived Cell Lines. Eur. J. Biochem..

[94] Izumi M., Yatagai F., Hanaoka F. (2004). Localization of Human Mcm10 Is Spatially and Temporally Regulated during the S Phase. J. Biol. Chem..

[95] Pollok S., Bauerschmidt C., Sänger J., Nasheuer H.-P.P., Grosse F. (2007). Human Cdc45 Is a Proliferation-Associated Antigen. FEBS J..

[96] Takaya J., Kusunoki S., Ishimi Y. (2013). Protein Interaction and Cellular Localization of Human CDC45. J. Biochem..

[97] Foertsch F., Kache T., Drube S., Biskup C., Nasheuer H.P., Melle C. (2019). Determination of the Number of RAD51 Molecules in Different Human Cell Lines. Cell Cycle.

[98] Huang J.-W., Taglialatela A., Acharya A., Leuzzi G., Nambiar T.S., Cuella-Martin R., Hayward S.B., Brunette G.J., Anand R., Soni R.K. (2019). Identification of MCM8IP, an Interactor of MCM8-9 and RPA1 That Promotes Homologous Recombination and DNA Synthesis in Response to DNA Damage. bioRxiv.

[99] Le T.T., Wu M., Lee J.H., Bhatt N., Inman J.T., Berger J.M., Wang M.D. (2023). Etoposide Promotes DNA Loop Trapping and Barrier Formation by Topoisomerase II. Nat. Chem. Biol..

[100] Van Ravenstein S.X., Mehta K.P., Kavlashvili T., Byl J.A.W., Zhao R., Osheroff N., Cortez D., Dewar J.M. (2022). Topoisomerase II Poisons Inhibit Vertebrate DNA Replication through Distinct Mechanisms. EMBO J..

[101] Yan H., Tammaro M., Liao S. (2016). Collision of Trapped Topoisomerase 2 with Transcription and Replication: Generation and Repair of DNA Double-Strand Breaks with 5′ Adducts. Genes.

[102] Lord C.J., Ashworth A. (2017). PARP Inhibitors: Synthetic Lethality in the Clinic. Science.

[103] Pommier Y., O’Connor M.J., De Bono J. (2016). Laying a Trap to Kill Cancer Cells: PARP Inhibitors and Their Mechanisms of Action. Sci. Transl. Med..

[104] Semlow D.R., Walter J.C. (2021). Mechanisms of Vertebrate DNA Interstrand Cross-Link Repair. Annu. Rev. Biochem..

[105] Reilly N.M., Yard B.D., Pittman D.L. (2019). Homologous Recombination-Mediated DNA Repair and Implications for Clinical Treatment of Repair Defective Cancers. Methods Mol. Biol..

[106] Huang J., Liu S., Bellani M.A., Thazhathveetil A.K., Ling C., deWinter J.P., Wang Y., Wang W., Seidman M.M. (2013). The DNA Translocase FANCM/MHF Promotes Replication Traverse of DNA Interstrand Crosslinks. Mol. Cell.

[107] Zhang J., Bellani M.A., Huang J., James R.C., Pokharel D., Gichimu J., Gali H., Stewart G., Seidman M.M. (2021). Replication of the Mammalian Genome by Replisomes Specific for Euchromatin and Heterochromatin. Front. Cell Dev. Biol..

[108] González-Acosta D., Blanco-Romero E., Ubieto-Capella P., Mutreja K., Míguez S., Llanos S., García F., Muñoz J., Blanco L., Lopes M. (2021). PrimPol-Mediated Repriming Facilitates Replication Traverse of DNA Interstrand Crosslinks. EMBO J..

[109] Huang J., Zhang J., Bellani M.A., Pokharel D., Gichimu J., James R.C., Gali H., Ling C., Yan Z., Xu D. (2019). Remodeling of Interstrand Crosslink Proximal Replisomes Is Dependent on ATR, FANCM, and FANCD2. Cell Rep..

[110] Dungrawala H., Rose K.L., Bhat K.P., Mohni K.N., Glick G.G., Couch F.B., Cortez D. (2015). The Replication Checkpoint Prevents Two Types of Fork Collapse without Regulating Replisome Stability. Mol. Cell.

[111] Hutchins J.R.A., Traver S., Coulombe P., Peiffer I., Kitzmann M., Latreille D., Méchali M. (2016). Proteomic Data on the Nuclear Interactome of Human MCM9. Data Brief.

[112] Tomita Y., Imai K., Senju S., Irie A., Inoue M., Hayashida Y., Shiraishi K., Mori T., Daigo Y., Tsunoda T. (2011). A Novel Tumor-Associated Antigen, Cell Division Cycle 45-like Can Induce Cytotoxic T-Lymphocytes Reactive to Tumor Cells. Cancer Sci..

[113] Tkáč J., Xu G., Adhikary H., Young J.T.F., Gallo D., Escribano-Díaz C., Krietsch J., Orthwein A., Munro M., Sol W. (2016). HELB Is a Feedback Inhibitor of DNA End Resection. Mol. Cell.

[114] Drissi R., Dubois M.L., Douziech M., Boisvert F.M. (2015). Quantitative Proteomics Reveals Dynamic Interactions of the Minichromosome Maintenance Complex (MCM) in the Cellular Response to Etoposide Induced DNA Damage. Mol. Cell. Proteom..

[115] Dubois M.L., Bastin C., Lévesque D., Boisvert F.M. (2016). Comprehensive Characterization of Minichromosome Maintenance Complex (MCM) Protein Interactions Using Affinity and Proximity Purifications Coupled to Mass Spectrometry. J. Proteome Res..

[116] Dunker A.K., Silman I., Uversky V.N., Sussman J.L. (2008). Function and Structure of Inherently Disordered Proteins. Curr. Opin. Struct. Biol..

[117] Warren C., Shechter D. (2017). Fly Fishing for Histones: Catch and Release by Histone Chaperone Intrinsically Disordered Regions and Acidic Stretches. J. Mol. Biol..

[118] Fox S.J., Kannan S. (2017). Probing the Dynamics of Disorder. Prog. Biophys. Mol. Biol..

[119] Liu Z., Huang Y. (2014). Advantages of Proteins Being Disordered. Protein Sci..

[120] Olsen J.G., Teilum K., Kragelund B.B. (2017). Behaviour of Intrinsically Disordered Proteins in Protein-Protein Complexes with an Emphasis on Fuzziness. Cell. Mol. Life Sci..

[121] Bleichert F., Botchan M.R., Berger J.M. (2017). Mechanisms for Initiating Cellular DNA Replication. Science.

[122] Li Y., Araki H. (2013). Loading and Activation of DNA Replicative Helicases: The Key Step of Initiation of DNA Replication. Genes Cells.

[123] Wu X., Malkova A. (2021). Break-Induced Replication Mechanisms in Yeast and Mammals. Curr. Opin. Genet. Dev..

[124] Srinivasan S.V., Dominguez-Sola D., Wang L.C., Hyrien O., Gautier J. (2013). Cdc45 Is a Critical Effector of Myc-Dependent DNA Replication Stress. Cell Rep..

[125] Outwin E., Carpenter G., Bi W., Withers M.A., Lupski J.R., O’Driscoll M. (2011). Increased RPA1 Gene Dosage Affects Genomic Stability Potentially Contributing to 17p13.3 Duplication Syndrome. PLoS Genet..

[126] Flygare J., Fält S., Ottervald J., Castro J., Dackland Å.L., Hellgren D., Wennborg A. (2001). Effects of HsRad51 Overexpression on Cell Proliferation, Cell Cycle Progression, and Apoptosis. Exp. Cell Res..

[127] Auth T., Kunkel E., Grummt F. (2006). Interaction between HP1alpha and Replication Proteins in Mammalian Cells. Exp. Cell Res..

[128] Maslov A.Y., Bailey K.J., Mielnicki L.M., Freeland A.L., Sun X., Burhans W.C., Pruitt S.C. (2007). Stem/Progenitor Cell-Specific Enhanced Green Fluorescent Protein Expression Driven by the Endogenous Mcm2 Promoter. Stem Cells.

[129] Gong B., Ma M., Yang X., Xie W., Luo Y., Sun T. (2019). MCM5 Promotes Tumour Proliferation and Correlates with the Progression and Prognosis of Renal Cell Carcinoma. Int. Urol. Nephrol..

[130] Ueno M., Itoh M., Sugihara K., Asano M., Takakura N. (2009). Both Alleles of PSF1 Are Required for Maintenance of Pool Size of Immature Hematopoietic Cells and Acute Bone Marrow Regeneration. Blood.

[131] Leonhardt H., Rahn H.P., Weinzierl P., Sporbert A., Cremer T., Zink D., Cardoso M.C. (2000). Dynamics of DNA Replication Factories in Living Cells. J. Cell Biol..

[132] Burks L.M., Yin J., Plon S.E. (2007). Nuclear Import and Retention Domains in the Amino Terminus of RECQL4. Gene.

[133] Sokka M., Rilla K., Miinalainen I., Pospiech H., Syväoja J.E. (2015). High Levels of TopBP1 Induce ATR-Dependent Shut-down of rRNA Transcription and Nucleolar Segregation. Nucleic Acids Res..

[134] Helbling-Leclerc A., Dessarps-Freichey F., Evrard C., Rosselli F. (2019). Fanconi Anemia Proteins Counteract the Implementation of the Oncogene-Induced Senescence Program. Sci. Rep..

[135] Kais Z., Rondinelli B., Holmes A., O’Leary C., Kozono D., D’Andrea A.D., Ceccaldi R. (2016). FANCD2 Maintains Fork Stability in BRCA1/2-Deficient Tumors and Promotes Alternative End-Joining DNA Repair. Cell Rep..

[136] Näf D., Kupfer G.M., Suliman A., Lambert K., D’Andrea A.D. (1998). Functional Activity of the Fanconi Anemia Protein FAA Requires FAC Binding and Nuclear Localization. Mol. Cell. Biol..

[137] Youssoufian H. (1996). Cytoplasmic Localization of FAC Is Essential for the Correction of a Prerepair Defect in Fanconi Anemia Group C Cells. J. Clin. Investig..

[138] Dodson G.E., Shi Y., Tibbetts R.S. (2004). DNA Replication Defects, Spontaneous DNA Damage, and ATM-Dependent Checkpoint Activation in Replication Protein A-Deficient Cells. J. Biol. Chem..

[139] Haring S.J., Mason A.C., Binz S.K., Wold M.S. (2008). Cellular Functions of Human RPA1. Multiple Roles of Domains in Replication, Repair, and Checkpoints. J. Biol. Chem..

[140] Spatafora J.W., Aime M.C., Grigoriev I.V., Martin F., Stajich J.E., Blackwell M. (2017). The Fungal Tree of Life: From Molecular Systematics to Genome-Scale Phylogenies. The Fungal Kingdom.

[141] Pennisi E. (2003). Drafting a Tree. Science.

[142] Uhlen M., Zhang C., Lee S., Sjöstedt E., Fagerberg L., Bidkhori G., Benfeitas R., Arif M., Liu Z., Edfors F. (2017). A Pathology Atlas of the Human Cancer Transcriptome. Science.

