# Supplementary material for: HROB Is Implicated in DNA Replication
Source: Genes (Basel). 2024 Dec 10;15(12):1587. doi: 10.3390/genes15121587 (PMC11675949; doi:10.3390/genes15121587)
Supplement: Supplementary file 1 [file genes-15-01587-s001.zip › genes-3309048-supplementary.pdf]

Supplementary Information

HROB is implicated in DNA replication.
Julia Kutz, Hannes Schmietendorf, Sheikh Anika Rahman, Franz Opel, Helmut Pospiech

Supplementary Material and Methods

Confocal laser scanning microscopy

Cells grown on glass cover slips were washed three times with PBS, fixed with 4% formaldehyde (Carl Roth, Karlsruhe, Germany, #4980) in PBS and again washed with PBS for three times. For DAPI staining, the cells were mounted in ProLong Gold antifade with DAPI (Invitrogen, Eugene, OR, USA, #P36931). For TO-PRO3 staining, the cells were permeabilized with 0.25% Triton X-100 (Carl Roth, #3051) in PBS for 5 min at room temperature followed by three times washing with PBS and incubation with 2 µM TO-PRO-3 iodide (Life technologies, Eugene, OR, USA, #T3605) in PBS for 12 min at room temperature. The cells were washed three times with PBS and mounted in ProLong Gold antifade (Invitrogen, #P36930). Fluorescence microscopy was performed using a LSM 710 Confocor3 microscope (Carl Zeiss, Jena, Germany) equipped with a Plan-Apochromat 63x/1.40 Oil DIC M27 objective, operated with the Zen 2.3 SP1 FP1 software (v14.0.12.201). Venus fusion proteins were excited using the 514 nm laser line of a 25 mW Argon/2-laser, Cerulean fusions with the 458 nm laser line of the same laser, DAPI with a 405 nm diode laser and TO-PRO-3 with a 633 nm diode laser. The detection pinhole was set to 1.00 airy unit (AU) for Venus, 2.40 AU for Cerulean, 2.79 AU for DAPI and 1.35 AU for TO-PRO-3. The main beam splitters (MBS) used and the detected wavelength ranges are as follows: Venus – MBS 458/514, 519-620 nm; Cerulean – MBS 458; 463-580 nm; DAPI – 410-507 nm; TO-PRO-3 – MBS 488/561/633; 638-759 nm.

HROB purification and estimation of the molecular number per cell

N-terminally 6xHis-tagged HROB was expressed and purified as follows: *E. coli* (BL21[DE3]) cells carrying the pRSF-Duet-6His-C17orf53 plasmid were grown at 37 °C in 1 l LB medium supplemented with 30 µg/ml kanamycin until an OD_{595nm} of 0.5 was reached. After induction of protein expression with 0.5 mM isopropyl-β-D-thiogalactopyranoside for 5 h at 25 °C, cells were harvested, washed with cold PBS, and resuspended into 15 ml PBS supplemented with 1 mg/ml lysozyme followed by incubation for 30 min on ice. Cells were disrupted by 24 × 10 s ultrasound pulses at 30% amplitude interrupted by 50 s incubations on ice (Sonifier W-450D, Branson Ultrasonics, Danbury, CT, USA). Triton X-100 (final concentration of 3%, Carl Roth) and protease inhibitors (1x cOmplete EDTA-free, RSartorius
oche, Mannheim, Germany) were added, and after rotation at 4 °C for 30 min and centrifugation at 110,000 g for 30 min, the supernatant was added to 690 ml buffer A (20 mM HEPES, pH 8.0). Further purification was performed using the Äkta avant 25 purification system (GE Healthcare, München, Germany) and the following protocol: Q sepharose fast flow chromatography (5 cm/160 ml column, 3 ml/min), washing with 5 column volumes of buffer A, elution in 5 column volumes linear gradient of buffers A and B (20 mM HEPES, 1 M NaCl, 240 mg/l Pefabloc SC inhibitors [Roche], pH 8.0), combination of HROB containing fractions identified via Western blot, Ni^{2+} sepharose fast flow chromatography (1.6 cm/8.4 ml column, 3 ml/min), washing with 5 column volumes of buffer C (20 mM HEPES, 0.5 M NaCl, 0.5 M guanidine hydrochloride, 20 mM imidazole, pH 7.4), elution in 10 column volumes linear gradient of buffers C and D (20 mM HEPES, 0.5 M NaCl, 0.5 M guanidine hydrochloride, 0.5 M imidazole, pH 7.4), combination of HROB containing fractions identified via Western blot, concentration to a final volume of 2 ml using a Vivaspin concentrator (10 kDa MWCO, Sartorius, Göttingen, Germany), Superdex200 HR 16/60 chromatography (1.6 cm diameter/60 cm length, 0.1 ml/min), elution in 120 ml buffer E (20 mM HEPES, 0.5 M NaCl, pH 7.4) in 1 ml fractions, combination of HROB containing fractions identified via Western blot, concentration to a final volume of 1 ml (Vivaspin, 10 kDa MWCO, Sartorius). Analysis of the resulting HROB SDS-PAGE band was performed using both, Western blot as well as mass spectrometric analysis (sample preparation: alkylation, tryptic digestion, extraction; nanoLC-ESI-MS [nanoAcquity-HPLC byWaters, Eschborn, Germany and Orbitrap Fusion Lumos by Thermo Fisher Scientific, Wilmigton, NC, USA], data dependend acquisition) of the excised band. The latter showed a contamination of the HROB containing band with *E. coli* proteins, suggesting a HROB content of 50%. Protein concentration was calculated after separation of the purified protein and an BSA standard curve (100 – 600 ng) in SDS-PAGE, followed by PageBlue staining (Thermo Fisher Scientific, Rockford, IL, USA) and band quantification using Image J (V 1.52n, Wayne Rasband, National Institutes of Health, Bethesda, MD, USA, https://imagej.net/ij/, 1997-2018). For estimation of the number of HROB molecules per cell, Western blot with full extracts of different cell numbers (1-2 x 10^5) and a HROB standard curve (0.39-3.10 ng) was performed. By quantifying the bands using Image J and creating a calibration line, the number of molecules per cell could then be calculated using the Avogadro constant and the HROB molecular weight of 69.6 kDa.

Supplementary Figures and Figure Legends

Supplementary Figure S1

A

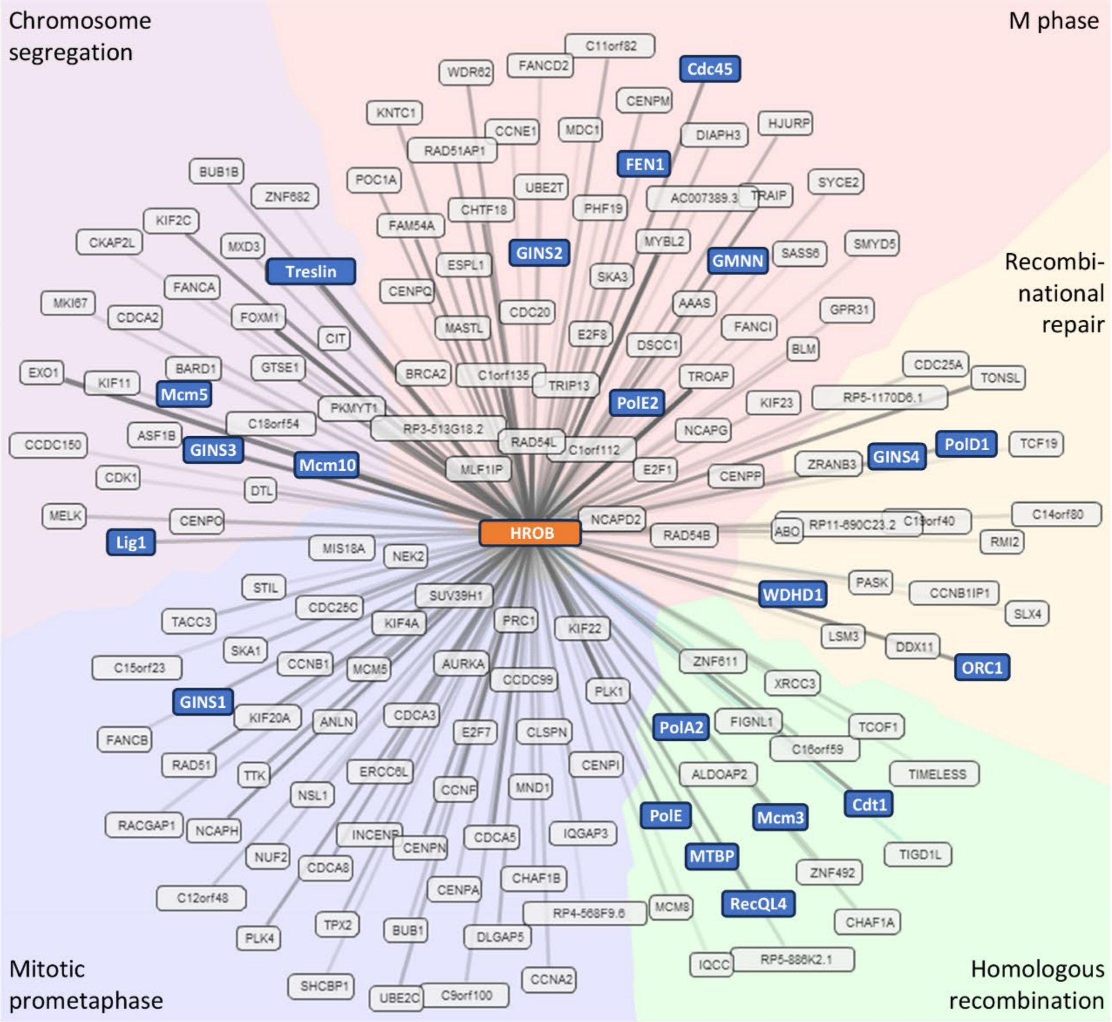

B

Pathway database	Biological process	P value
Gene Ontology Biological Process (GO biological process)	M phase	$1.13 \cdot 10^{-18}$
	Chromosome segregation	$1.18 \cdot 10^{-18}$
	Recombinational repair	$1.86 \cdot 10^{-18}$
	Homologous recombination	$5.24 \cdot 10^{-18}$
	Prometaphase	$7.29 \cdot 10^{-18}$
	DNA replication	$4.98 \cdot 10^{-17}$
Kyoto Encyclopedia of Genes and Genomes (KEGG)	Homologous recombination	$2.21 \cdot 10^{-21}$
	Base excision repair	$9.76 \cdot 10^{-12}$
	Cell cycle	$1.24 \cdot 10^{-11}$
	DNA replication	$1.71 \cdot 10^{-11}$
	Mismatch repair	$3.43 \cdot 10^{-10}$
	Nucleotide excision repair	$2.98 \cdot 10^{-5}$
Reactome Pathway Database	DNA replication	$2.24 \cdot 10^{-18}$
	Mitotic cell cycle	$2.44 \cdot 10^{-17}$
	G2/M checkpoints	$5.44 \cdot 10^{-17}$
	Prometaphase	$9.81 \cdot 10^{-16}$
	M phase	$1.01 \cdot 10^{-16}$
	Cell cycle	$1.63 \cdot 10^{-16}$

Supplementary Figure S1. HROB shares expression regulation with several DNA replication proteins.

The HROB co-expression network (**A**) and the constitutive function prediction (**B**) was generated using GeneNetwork (version 1.0 [used microarray data, subsequent versions use RNA seq data] [68]). **A** DNA replication factors are marked by blue boxes. Thick gray connection lines indicate strong positive co-expression, thin connection lines indicate weak positive co-expression. The proteins were divided into groups according to their function as labelled and depicted by different background colours. **B** Overview of the top six HROB protein function predictions by GeneNetwork for the pathway databases indicated.

Supplementary Figure S2

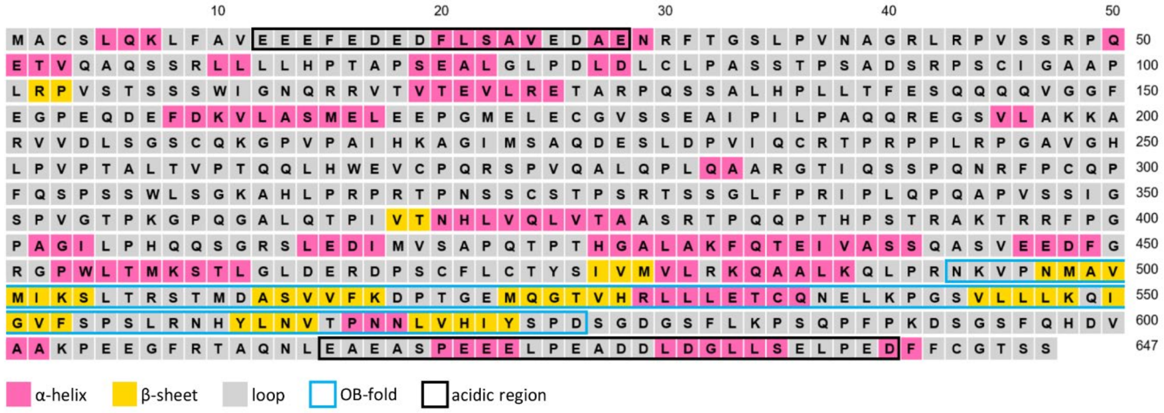

Supplementary Figure S2. Illustration of the secondary structure prediction of HROB. The structure prediction of human HROB was performed with PSIPRED (version 4.0, [62]). Sections predicted as α-helix are highlighted in pink, sections predicted as β-sheet are highlighted in yellow and other sections predicted as “loops” (disordered) are highlighted in gray. The OB-fold (oligonucleotide/oligosaccharide-binding fold motif) identified with HMMER (version 3.3, HMMSCAN against Pfam [64]) is outlined in blue. Protein regions with a bias for negatively charged amino acids are outlined in black. The numbers indicate the positions of amino acids.

Supplementary Figure S3

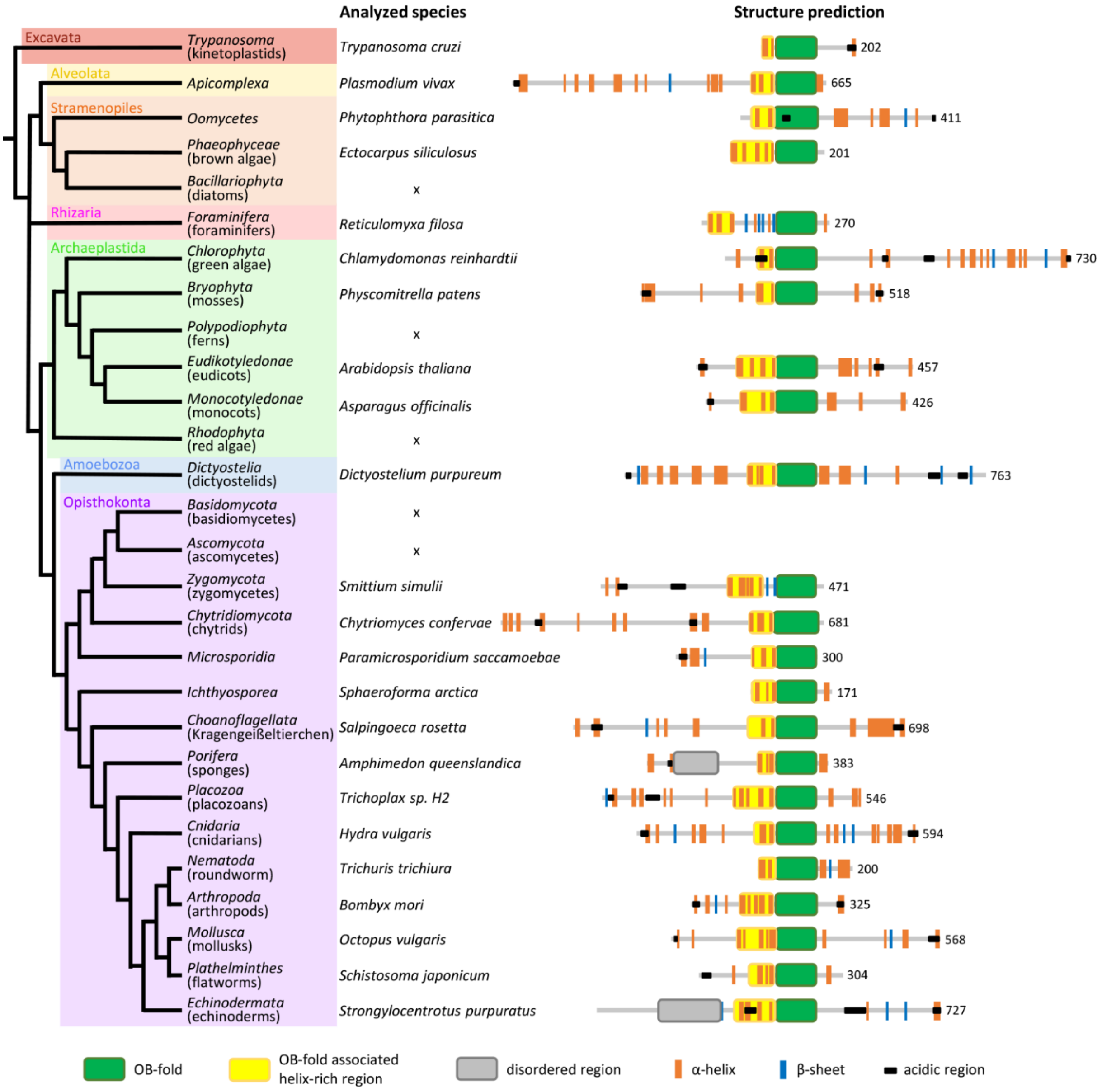

Supplementary Figure S3. The structure of HROB is conserved in eukaryotes. Identification of HROB sequence homologs was performed using Ensembl (version 99, [67]), Pfam (version 32.0, [65]) and chiefly NCBI blastp suite (PSI-BLAST, [66]). The structure prediction was performed using PSIPRED (version 4.0, [62]). The OB-folds (oligonucleotide/oligosaccharide-binding fold motif, green) were identified with HMMER (version 3.3, HMMSCAN using Pfam, [64]). α-Helices (orange) and β-sheets (blue) outside of the OB-fold were shown from a minimum length of 5 amino acids. OB-fold-associated helix-rich regions (yellow) were defined as sections with a proportion of at least 40% helices (independent of the length). Disordered regions (gray) consider at least 80 amino acid long, undisrupted sections without α-helix or β-sheet prediction. Acidic regions (black) were identified using SAPS [75] with manual post-selection of regions with at least 7 and at least 30% of negatively charged amino acids and without positively charged amino acids. The numbers indicate the lengths of the proteins. The taxonomic classification was performed by using NCBI Taxonomy (version 12/2019, [76]), Open Tree of Life (version 3.2, [76]) and for *Zygomycota* [133]. The protein-ID of all shown homologs are listed in Supplementary Table S3 (illustration inspired by [134]).

Supplementary Figure S4

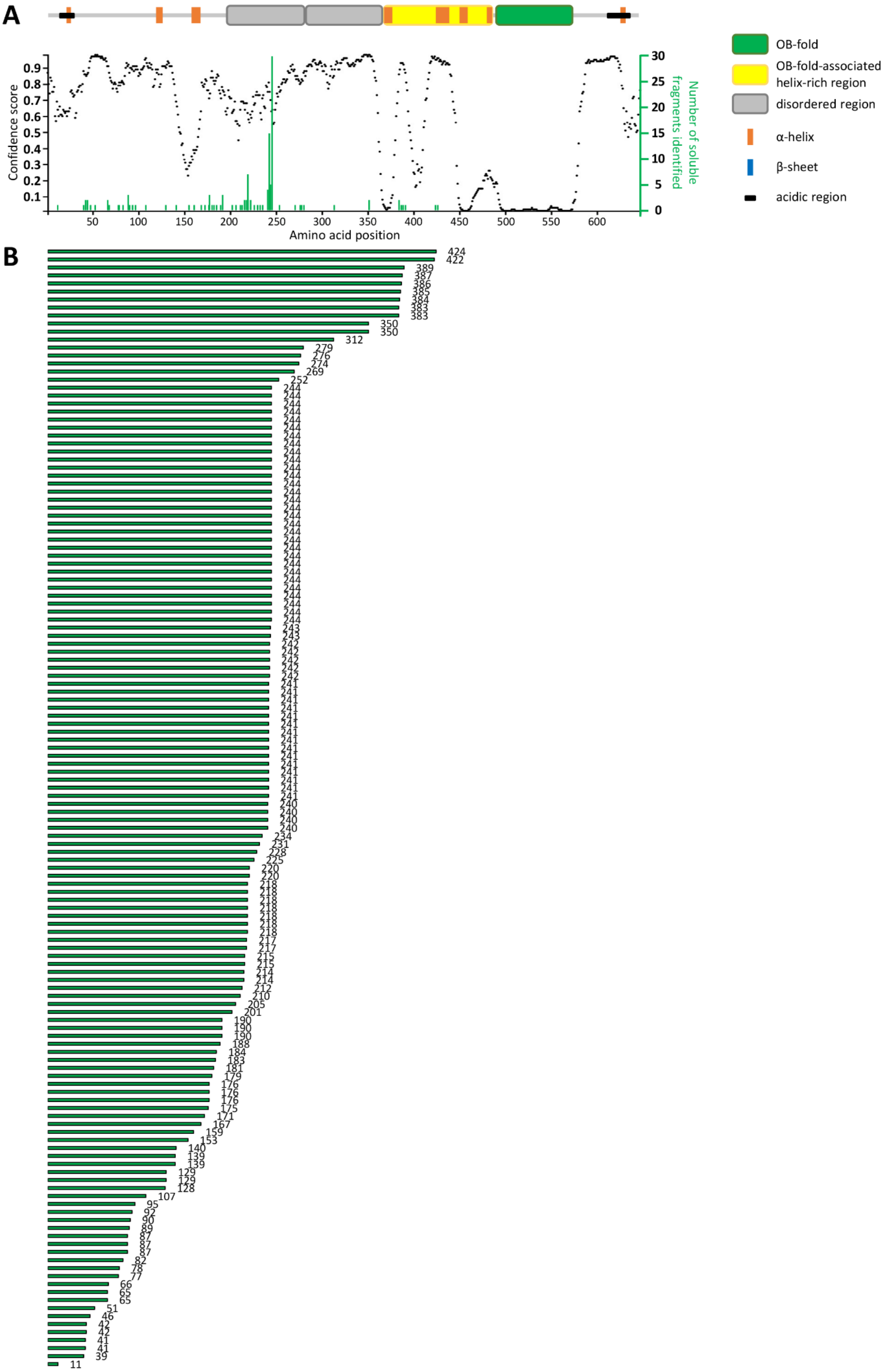

Supplementary Figure S4. Protein fragments identified by the Random PCR Screen are consistent with the HROB structure prediction.

A Scheme of the human HROB structure prediction (**top**) performed with PSIPRED (version 4.0, [62]) and of the intrinsic disorder prediction (**bottom**) created using DISOPRED (version 3, [63]). The OB-fold (oligonucleotide/oligosaccharide-binding fold motif, domain of unknown function 4539, N493-D576, green) was identified with HMMER (version 3.3, HMMSCAN using Pfam, [64]). α-Helices (orange) and β-sheets (blue) outside of the OB-fold were shown from a minimum length of 5 amino acids. OB-fold-associated helix-rich regions (yellow) were defined as sections with a proportion of at least 40% helices (independent of the length). Disordered regions (gray) consider at least 80 amino acid long, undisrupted sections without α-helix or β-sheet prediction. Acidic regions (black) were identified using SAPS [75] with manual post-selection of regions with at least 7 and at least 30% of negatively charged amino acids and without positively charged amino acids. The number and the lengths of soluble fragments identified using the random PCR screen are indicated in green within the intrinsic disorder diagram. **B** Graphical overview about all soluble HROB fragments identified by the random PCR screen. Green lines represent the lengths of the fragments in relation to the full-length protein shown in A. The numbers indicate the position of the last amino acid of each fragment.

Supplementary Figure S5

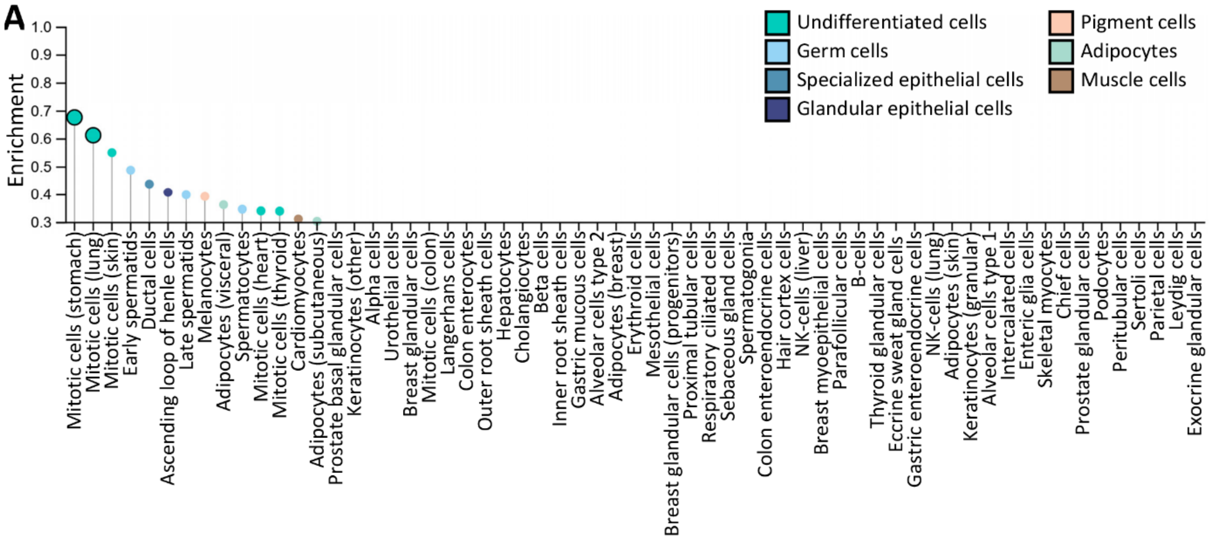

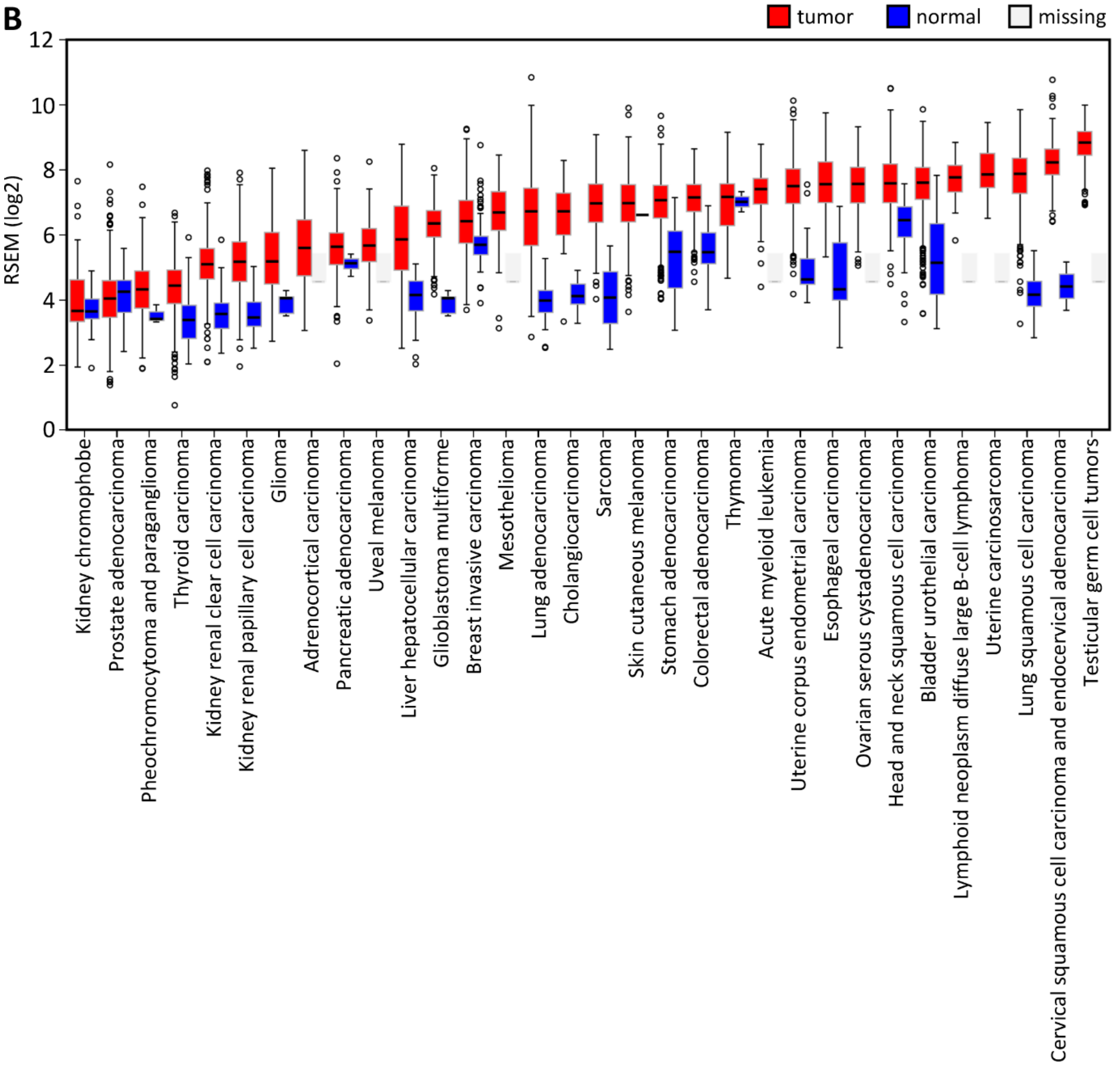

Supplementary Figure S5. The HROB protein level is upregulated in cancer and proliferating cells.

A The cell type specific expression prediction of human HROB based on an integrated network analysis of RNAseq data of unfractionated tissue samples is originated from The Human Protein Atlas tissue cell [81]. The plotted enrichment prediction scores are the mean correlations between HROB and three reference transcripts selected to represent each cell type profiled within the tissues. Enlarged, encircled symbols indicate the classification of HROB as cell type enriched in the corresponding cell types. **B** HROB expression in different cancers and the corresponding healthy tissues. The diagram was created by FireBrowse (version 1.1.40, Broad Institute TCGA Genome Data Analysis Center 2016). The quantification of the HROB expression via RSEM (RNA-Seq by expectation maximization) is shown for cancer tissue in red and healthy tissues in blue. Missing data for normal tissue are shown as gray boxes.

Supplementary Figure S6

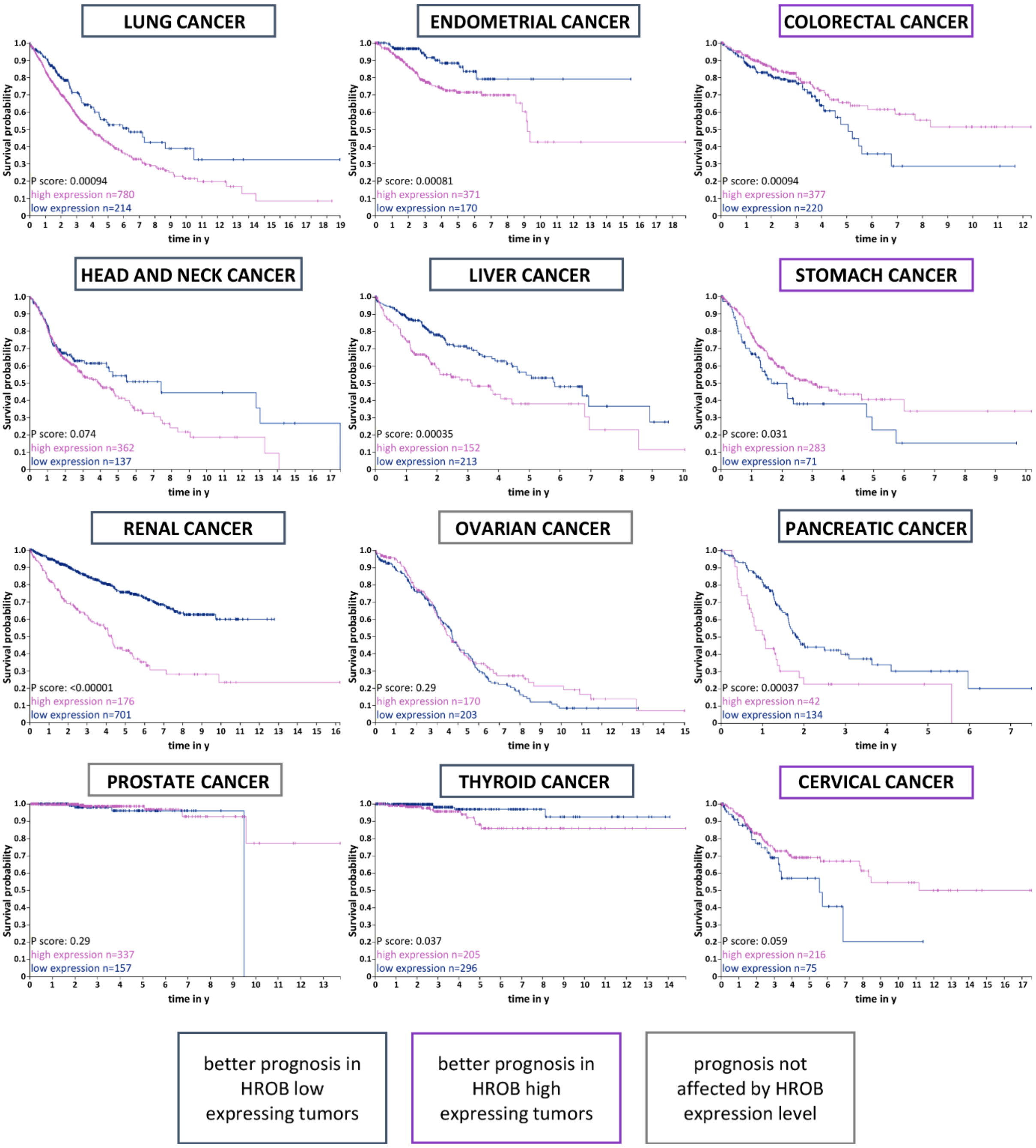

Supplementary Figure S6. Influence of the expression level of HROB on the probability of survival in different cancers.

The Kaplan-Meier diagrams for the indicated carcinogenic diseases were taken from The Human Protein Atlas pathology (version 19.1, [135]). The patient samples were divided into samples with high HROB expression (below the respective threshold value; shown in purple) and samples with low HROB expression (above the respective threshold value; shown in blue) based on the HROB mRNA level and an individual cut-off for each entity. The p values of the log-rank tests as well as the number of high and low expressing samples are shown in the respective diagrams.

Supplementary Figure S7

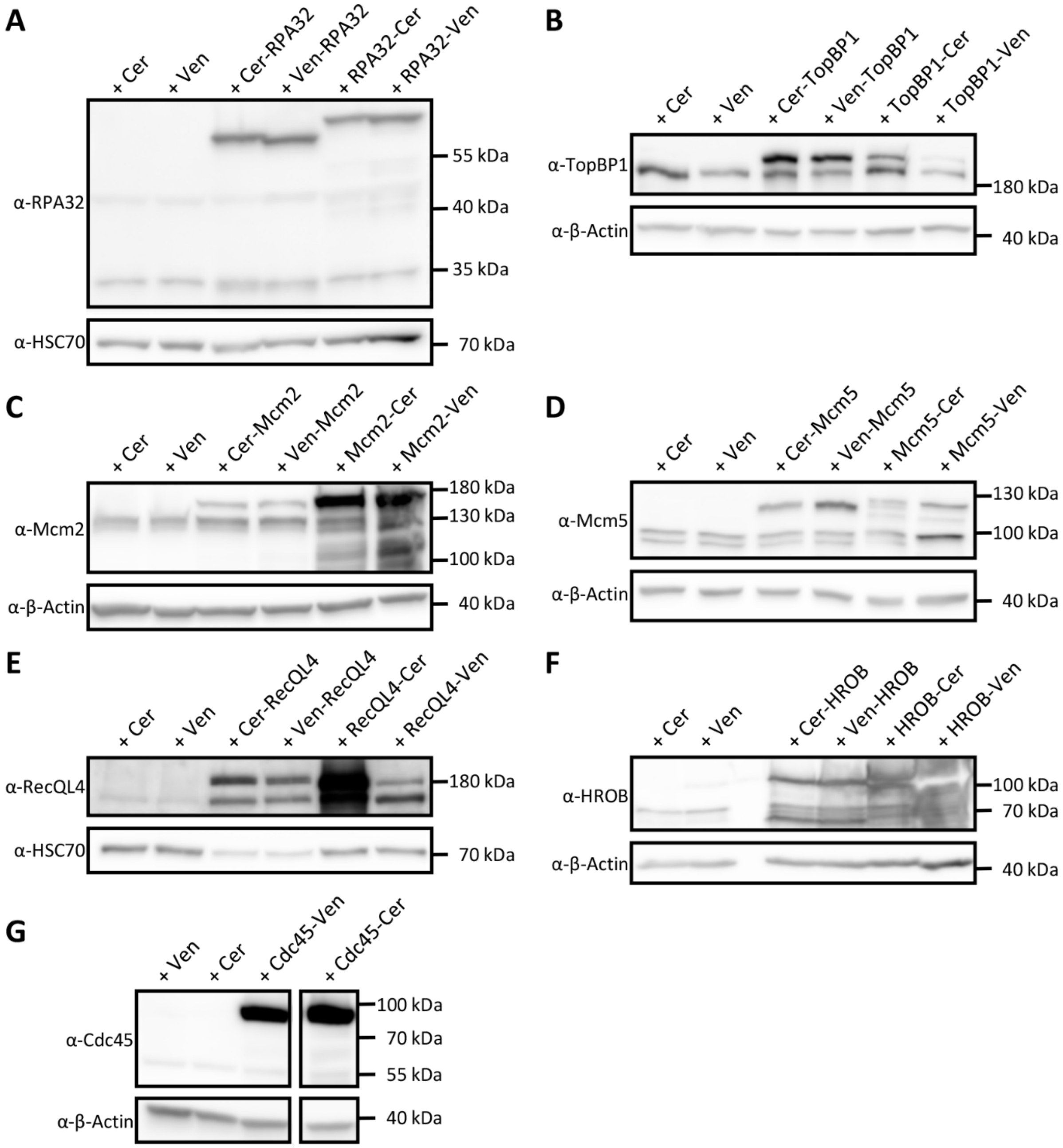

Supplementary Figure S7. Verification of the correct expression of the generated fluorescent fusion proteins.

HEp-2 cells were transiently transfected with plasmids coding for the fluorescence proteins Cerulean (Cer) or Venus (Ven) or with plasmids coding for RPA32 (**A**), TopBP1 (**B**), Mcm2 (**C**), Mcm5 (**D**), RecQL4 (**E**), HROB (**F**) or Cdc45 (**G**) tagged with Cer or Ven, respectively. One day after transfection, full cell extracts were prepared and used for Western blots (abbreviation: α – anti, n=1).

Supplementary Figure S8

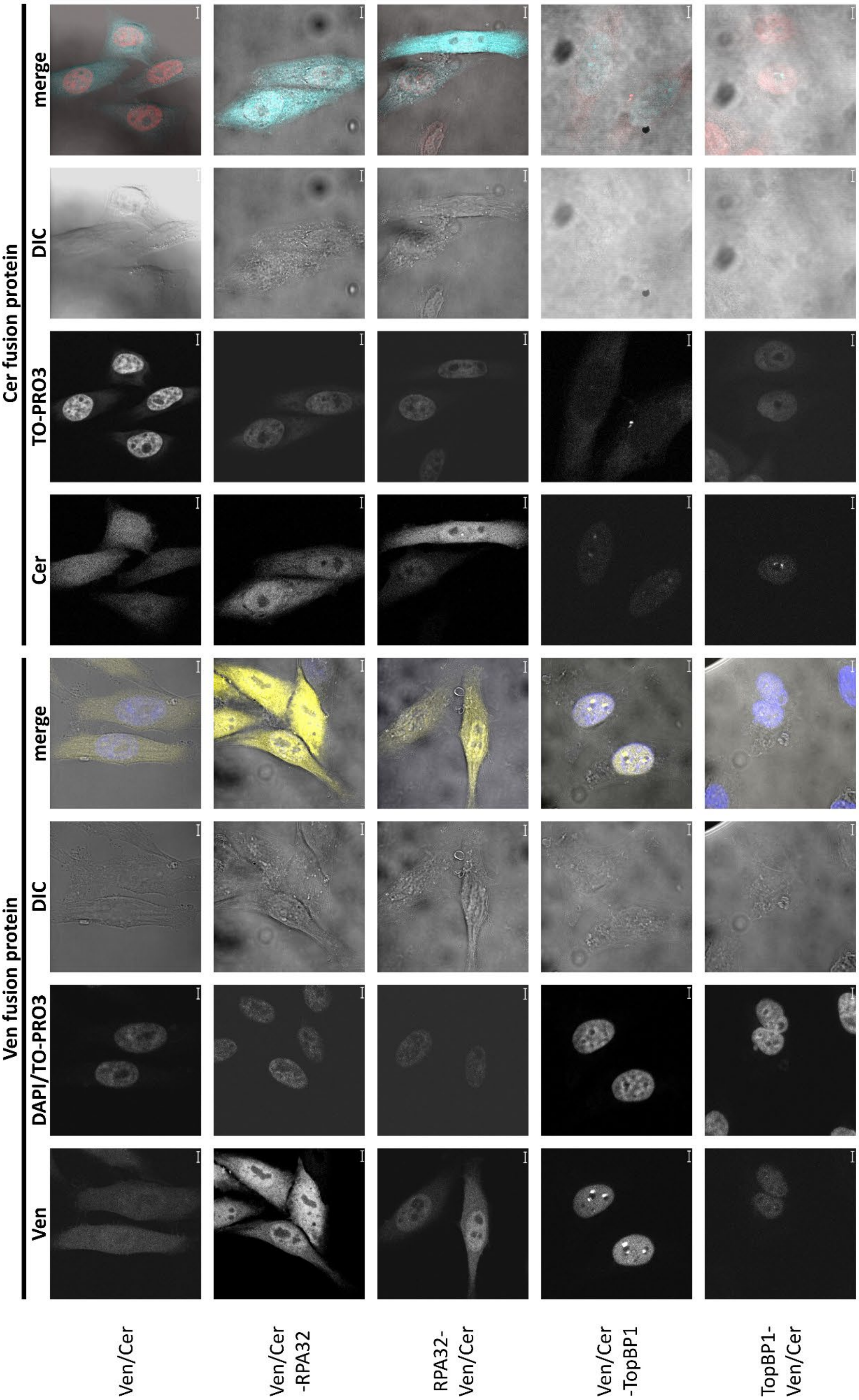

Supplementary Figure S8 (continued)

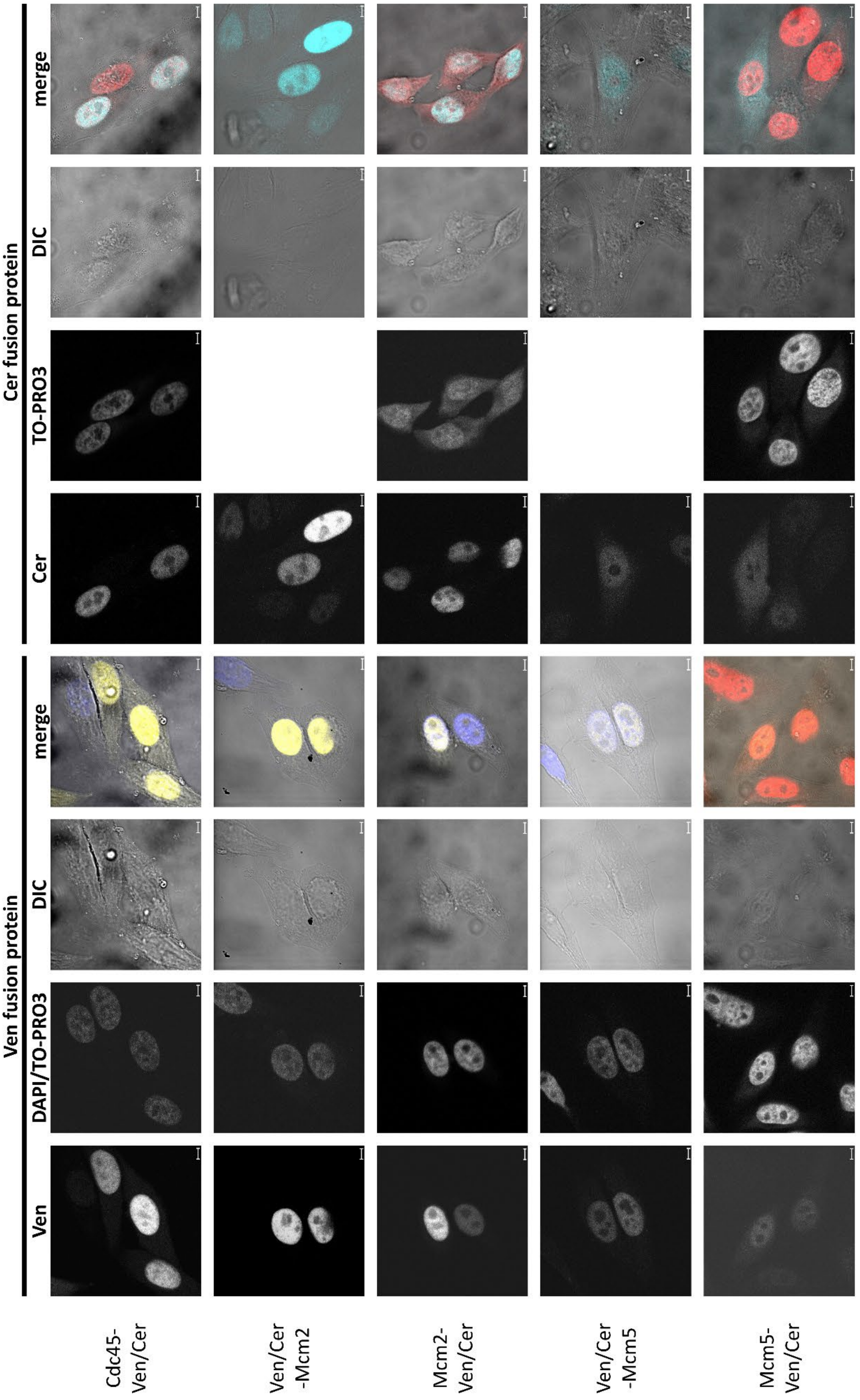

Supplementary Figure S8 (continued)

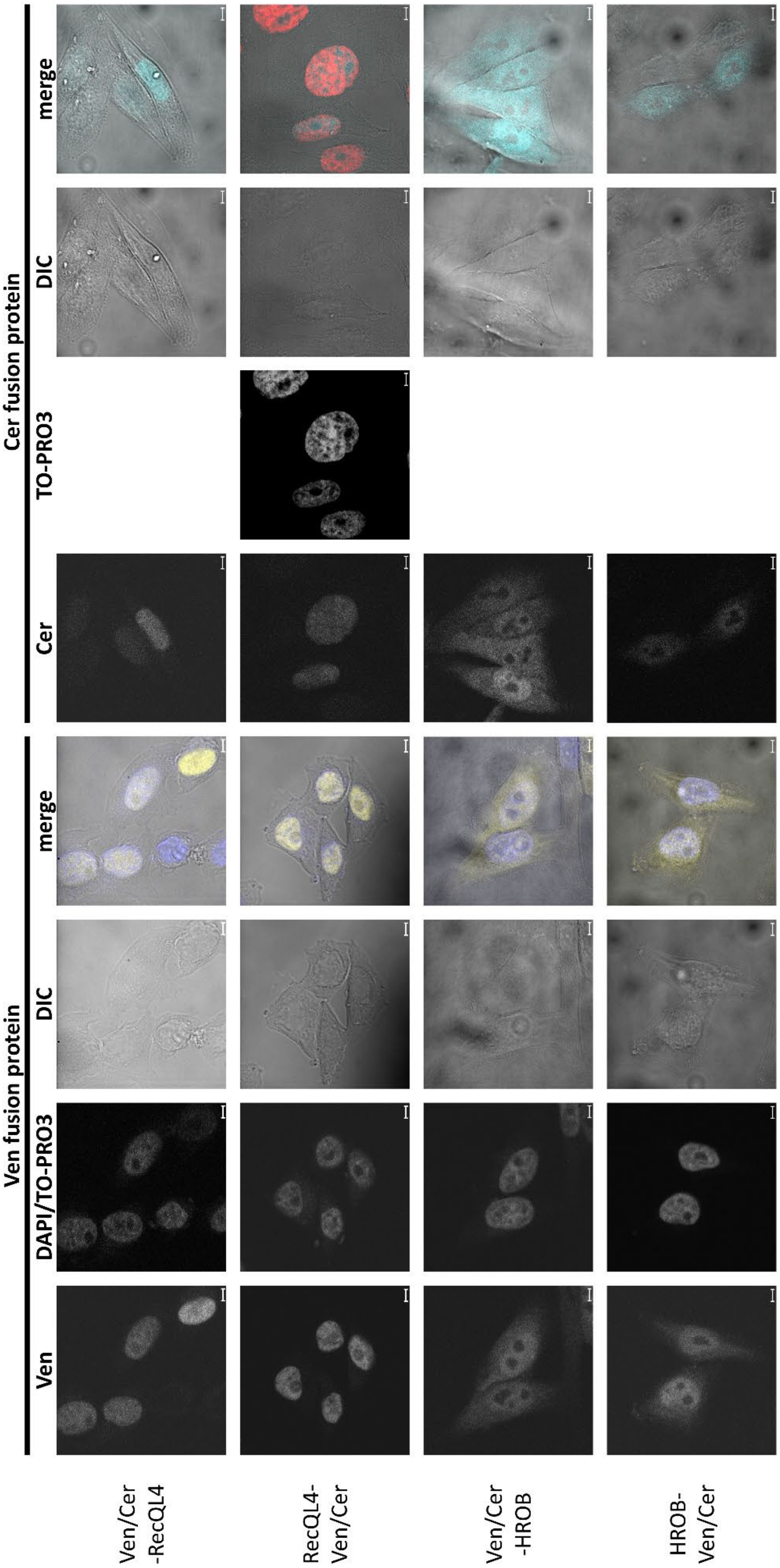

Supplementary Figure S8. Verification of the nuclear localization of the generated fluorescent fusion proteins.

HEp-2 cells were transiently transfected with plasmids coding for the fluorescence proteins Cerulean (Cer) or Venus (Ven) or with plasmids coding for Cdc45, RPA32, TopBP1, RecQL4, Mcm2, Mcm5 or HROB fused to Cer or Ven, respectively. One day after transfection, cells were fixed and analyzed via fluorescence microscopy. The nuclear localization was verified with DAPI or TO-PRO3 staining or via differential interference contrast (DIC). Within the merged pictures, Ven is shown in yellow, Cer in turquoise, DAPI in blue, TO-PRO3 in red and DIC in gray. Scale bar: 5 µm (n=2).

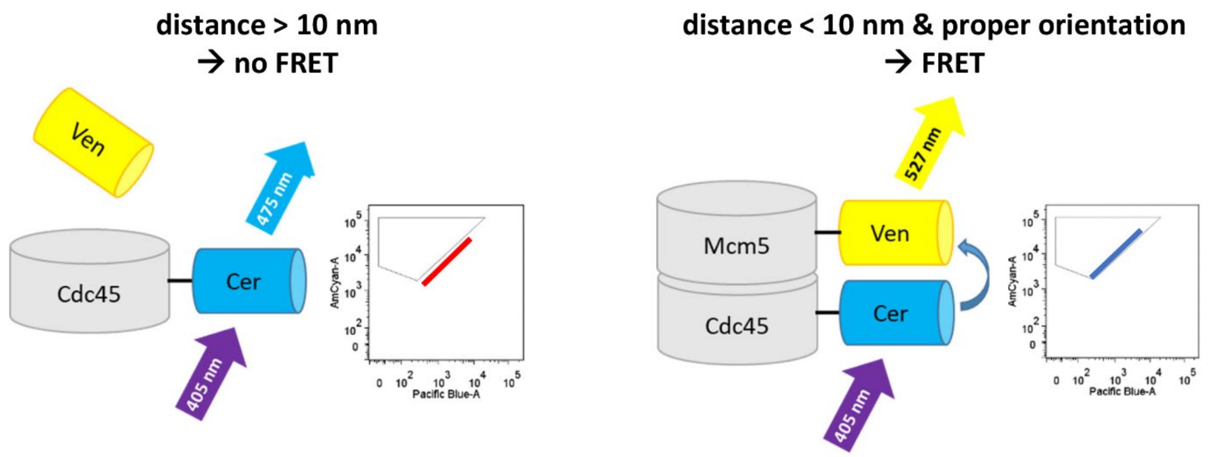

Supplementary Figure S9. The principle of the flow cytometry FRET assay.

Schematic overview of the principle of the FRET assay. The emission spectrum of Cer overlaps with the absorption spectrum of Ven, which allows a radiation free energy transfer (FRET) from Cer (donor) to Ven (acceptor) if the fluorophores are in close proximity and proper orientation to each other. In the cytometry FRET assay, the Cer within living cells co-expressing both fluorophores is excited using a 405 nm laser. If the fluorescent proteins within the cells do not FRET, the excitation results in fluorescence of Cer detectable within the Pacific Blue channel (405 nm laser, filter 448/45, mirror 448/45) of the cytometer. If the intracellular fluorescent proteins are in close proximity, FRET can occur: After the excitation of Cer the energy is transferred to Ven. The resulting fluorescence of Ven is detectable within the AmCyan channel (405 nm laser, filter 528/45, mirror 500 LP) and the consequently reduced intensity of the Cer fluorescence in the Pacific Blue channel of the cytometer.

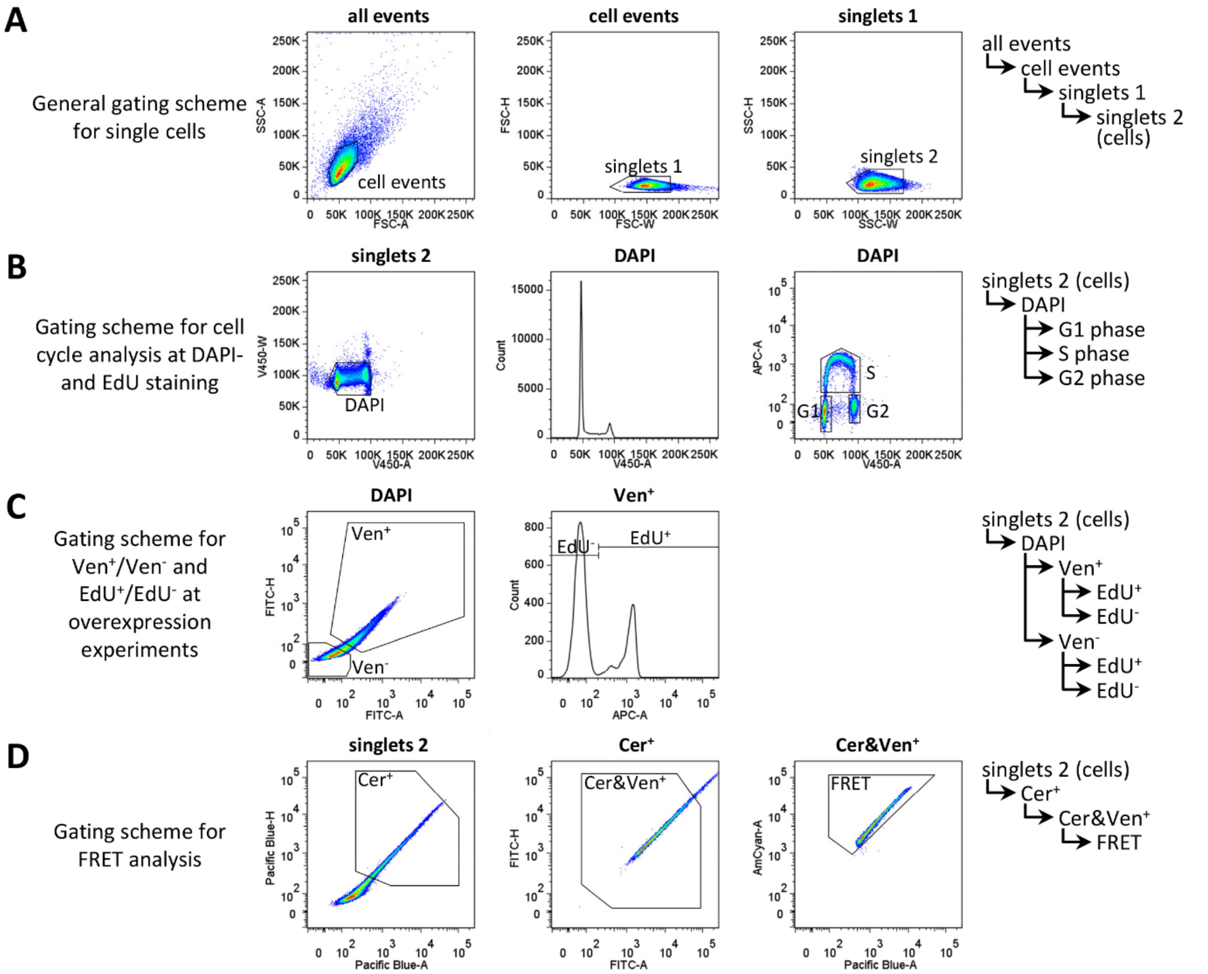

Supplementary Figure S10. Utilized gating schemes for the analysis of flow cytometric experiments.

The name of the initial population is shown above the diagram, the names of the newly selected subpopulations are shown within the diagram. The order of population selection is summarized in the right-handed sections. In principle, individual cells were first selected in three steps for all samples (**A**). These single cells were subsequently differentiated into the named subpopulations using the listed selection schemes for cell cycle analyses (**B**), overexpression analyses (**C**) and FRET experiments (**D**).

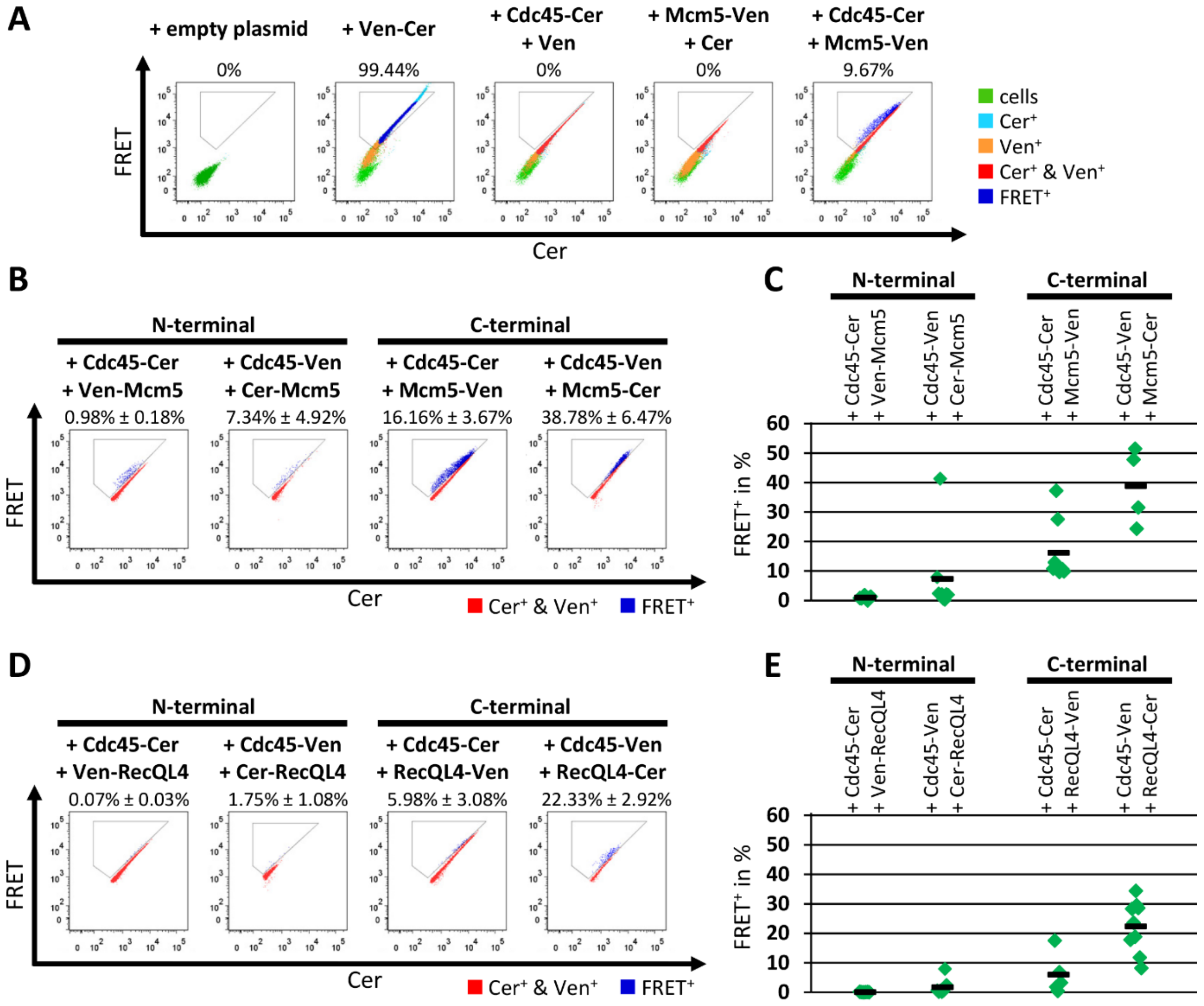

Supplementary Figure S11. The flow cytometry FRET assay.

For the FRET (Förster resonance energy transfer) assays, HEp-2 cells were transiently transfected with empty plasmid (pcDNA3.1+), plasmids coding for the fluorescence proteins Cerulean (Cer) or Venus (Ven) or with plasmids coding for fluorescent fusion proteins, followed by cytometric analysis. **A** The cytometry FRET assay was applied to Cdc45 and Mcm5, which are two DNA replication proteins showing a well-characterized interaction during replication. Representation of the sample set for each FRET experiment: Cells transfected with empty vector (pcDNA3.1+) served as a non-fluorescent control. Cells expressing the FRET-capable Ven-Cer fusion protein were used as positive control and cells transfected with not-fused Cer or Ven encoding plasmids in combination with a Cdc45 or Mcm5 fluorescent fusion protein were used as negative controls. The intensities in the FRET channel were plotted against the Cer intensities. The populations are overlapping in the order cells (green), Cer positive cells (Cer^+, turquoise), Ven positive cells (Ven^+, orange), Cer and Ven double positive cells (Cer^+ & Ven^+, red) and FRET positive cells ($FRET^+$, blue) are shown. The percentages indicate the respective proportion of $FRET^+$ in the sample shown (n=8). **B** Representative images of samples of cells co-expressing the indicated Cdc45 and Mcm5 fusion proteins. The intensities in the FRET channel were plotted against the Cer intensities. The populations are shown overlapping in the order "Cer and Ven double positive cells (Cer^+ & Ven^+, red) and FRET positive cells ($FRET^+$, blue). The percentages indicate the mean (± SEM) of the proportions of $FRET^+$ from at least four independent samples (n=4-8).**C** Diagram overview of the data shown in B (see Supplementary Table S6 for original data and Supplementary Table S4 for quantification). **D** Representative images of samples of cells co-expressing the indicated Cdc45 and RecQL4 fusion proteins. The intensities in the FRET channel were plotted against the Cer intensities. The populations are shown overlapping in the order "Cer and Ven double positive cells (Cer^+ & Ven^+, red) and FRET positive cells ($FRET^+$, blue). The percentages indicate the mean (± SEM) of the proportions of $FRET^+$ from at least five independent samples (n=5-9). **E** Quantification of the data shown in D (see also Supplementary Table S4 and S6).

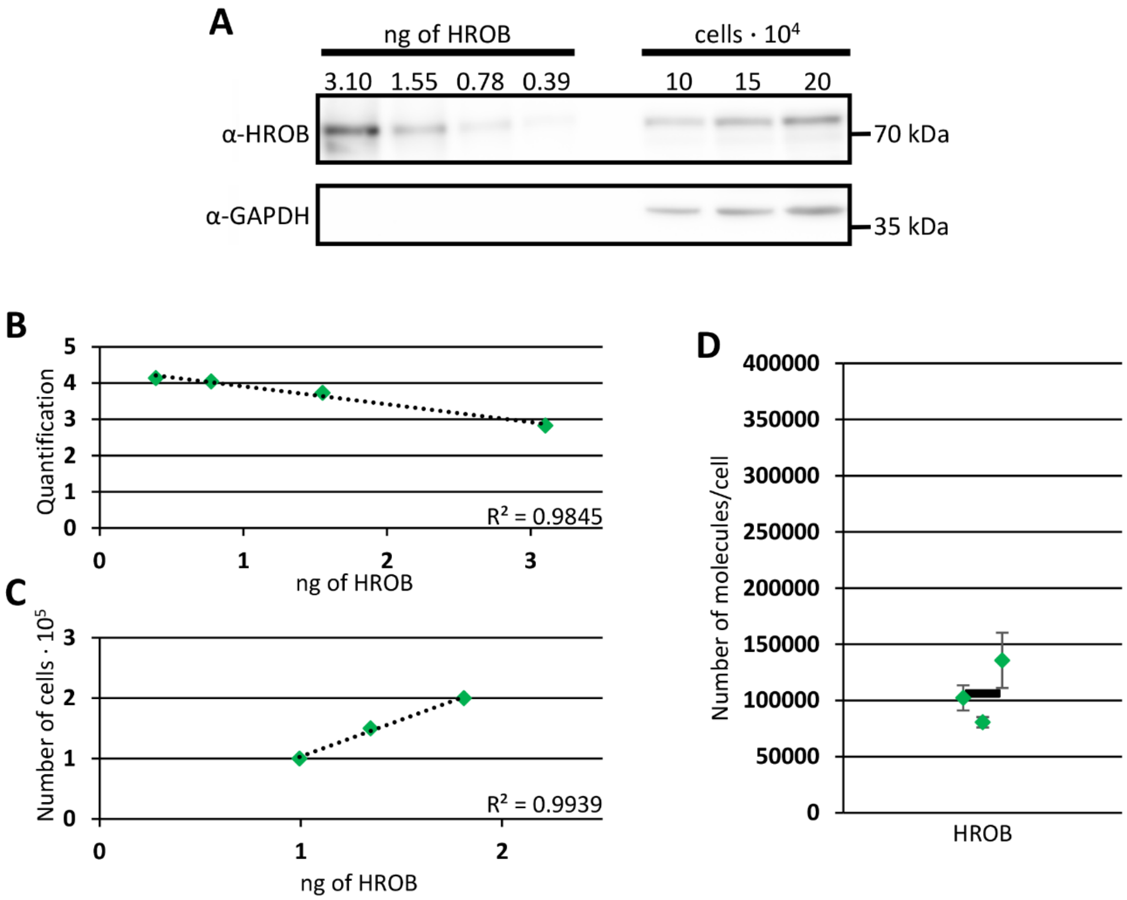

Supplementary Figure S12. Estimation of the number of HROB molecules per cell.

A The Western blot was performed using the indicated mass of purified HROB and full extracts of the indicated number of HEp-2 cells (abbreviation α – anti, n=3). **B** The calibration line was generated utilizing the quantified intensities of the HROB bands of the purified protein and the corresponding amount of applied HROB protein shown in A. **C** The calculation of the HROB amount in the cell extracts was performed using the quantified intensities of the HROB bands of the cell extracts shown in A and the calibration line shown in B. **D** Overview about the calculated number of HROB molecules per cell. Each data point represents an individual biological sample summarizing the three values of the three different cell numbers (indicated in A) used. The error bars represent the standard deviations. The mean is marked as black bar and indicates 106200 ± 13600 (± SEM) HROB molecules per cell.

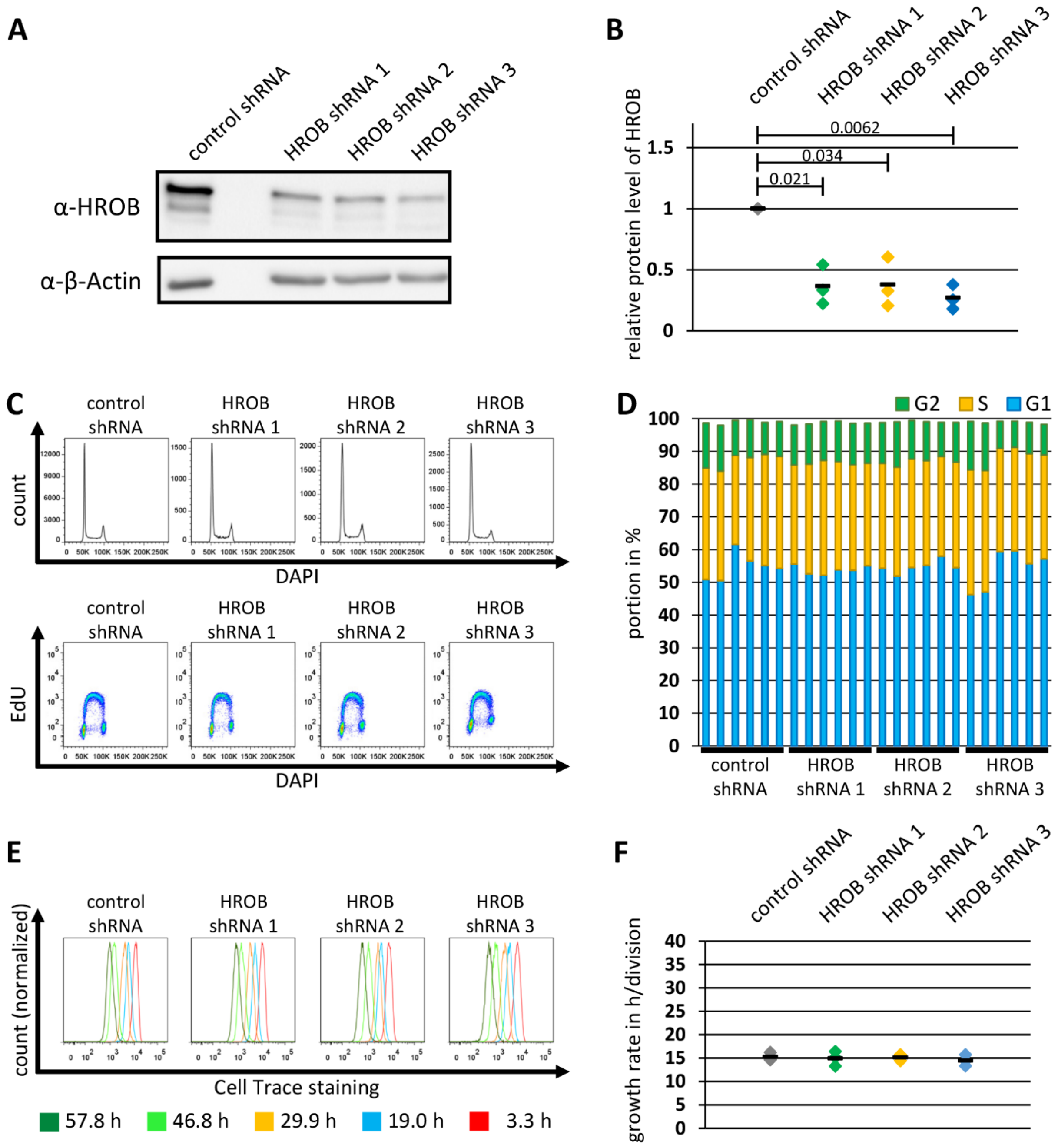

Supplementary Figure S13. Knock-down of HROB does not affect cell cycle distribution or growth rate.

HEp-2 cell lines expressing control shRNA or one of three different shRNAs directed against HROB were used. **A** Western blot was performed using full extracts of these cell lines (abbreviation α – anti, n=3). **B** The signal intensities of the HROB Western blot protein bands shown in A were quantified and normalized. Each data point represents an independent biological sample. The means are represented by black bars. P values were determined using two-sided Student's *t*-tests. **C-D** The cell cycle distribution of these cells was analyzed via flow cytometry after EdU and DAPI staining (n=6). **C** Representative images of the cell cycle analysis diagrams. **D** Representation of the values determined for the percentages of the cell cycle phases. **E-F** These cells were treated with Cell Trace dye to determine the growth rate and analyzed cytometrically for the dye at the indicated time points 3.3-57.8 h after staining (n=4). **E** Images of the Cell Trace staining analysis diagrams. **F** The growth rates of the cell lines were determined based on the cell trace intensities. Each data point represents an independent experiment. The means are represented by black bars.

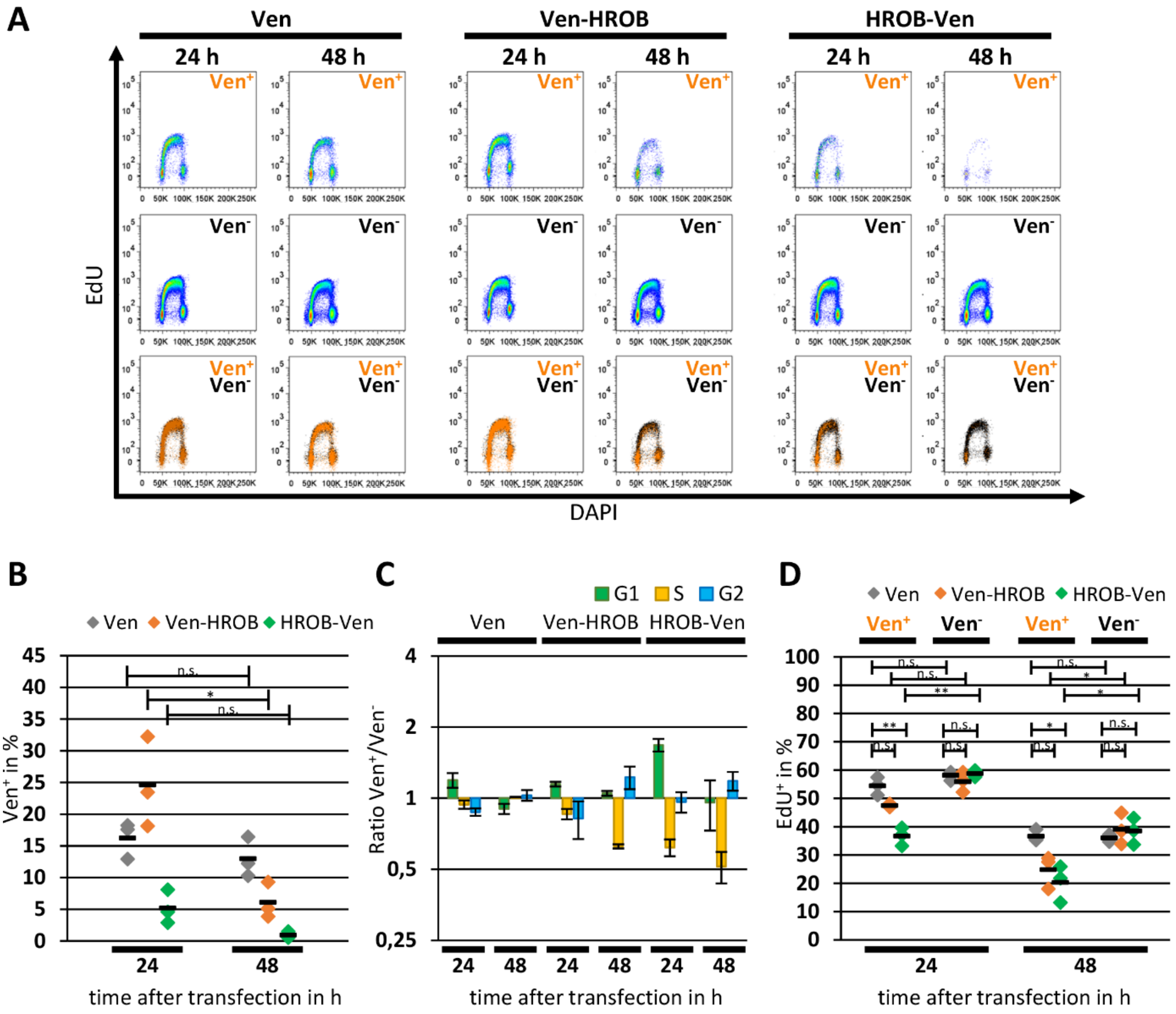

Supplementary Figure S14. HROB-overexpressing U2OS cells show an S phase depletion. The results shown in Figure 5 using HEp-2 cells were confirmed using another cell line. U2OS cells were transiently transfected with plasmids encoding Venus (Ven), Ven-HROB or HROB-Ven. 24 h or 48 h after transfection, cell cycle was analyzed via flow cytometry after EdU and DAPI staining. For each sample, Ven positive (Ven^+) and Ven negative (Ven^-) cells were distinguished and the proportion of EdU positive (EdU^+) cells was determined (n=3). **A** Representative images of the cell cycle analysis diagrams. The EdU intensities were plotted against the DAPI intensities. The upper row shows Ven^+ cells and the middle row Ven^- cells. Ven^+ (in orange) and Ven^- (in black) cells are overlayed within the diagrams of the bottom row. **B** The cells were analyzed cytometrically for the proportion of Ven^+ cells. Each data point represents an independent biological sample. The means are represented by black bars. P values (*p < 0.05, **p < 0.01, ***p < 0.001) were determined using two-sided Student's *t*-tests without corrections for multiple testing. **C** The cell cycle distribution of the Ven^+ and Ven^- cells was determined. The means of the ratios of the values for the percentages of G1 (in green), S (in yellow) and G2 phase (in blue) are shown. The error bars indicate the standard deviations. The information on significance was omitted for reasons of clarity. **D** The Ven^+ and Ven^- cells were cytometrically analyzed for the proportion of EdU^+. Each data point represents an independent biological sample. The means are represented by black bars. P values (*p < 0.05, **p < 0.01, ***p < 0.001) were determined using Student's *t*-tests without corrections for multiple testing.

Supplementary Tables

Supplementary Table S1: List of primers used for the generation of plasmids

Generated plasmids	Primers	Restriction sites
pmCer-C17orf53 and pmVen-C17orf53	5' GACGAATTCTATGGCGTGCAGTTTGCAG 3'<br>5' CGCGTCGACTCAACTACTGGTCCCACAGAAG 3'	*Eco*RI, *Sal*I
pcDNA3-C17orf53-mCer and pcDNA3-C17orf53-mVen	5' GACGAATTCTATGGCGTGCAGTTTGCAG 3'<br>5' GGACTCGAGACTACTGGTCCCACAGAAGAAG 3'	*Eco*RI, *Xho*I
pcDNA3-Cdc45-mCer and pcDNA3-Cdc45-mVen	5' GACGGATCCGCCACCATGTTCGTGTCCGATTTCC 3'<br>5' GACCTCGAGGGACAGGAGGGAAATAAG 3'	*Bam*HI, *Xho*I
pmCer-Mcm2 and pmVen-Mcm2	5' CGGAATTCGATGGCGGAATCATCGGAATC 3'<br>5' GCGTGGATCCTCAGAACTGCTGCAGGATC 3'	*Eco*RI, *Bam*HI
pcDNA3-Mcm2-mCer and pcDNA3-Mcm2-mVen	5' CGGAATTCGATGGCGGAATCATCGGAATC 3'<br>5' GAGCGGCCGCAAGAACTGCTGCAGGATCA 3'	*Eco*RI, *Not*I
pmCer-Mcm5 and pmVen-Mcm5	5' CGGAATTCGATGTCGGGATTCGACGATCC 3'<br>5' CTGGATCCTCACTTGAGGCGGTAGAGAAC 3'	*Eco*RI, *Bam*HI
pcDNA3-Mcm5-mCer and pcDNA3-Mcm5-mVen	5' CGGAATTCGATGTCGGGATTCGACGATCC 3'<br>5' GAGCGGCCGCATCTTGAGGCGGTAGAGAACCTTGC 3'	*Eco*RI, *Not*I
pmCer-RecQL4 and pmVen-RecQL4	5' CTAGATCTATGGAGCGGCTGCGGGACGTG 3'<br>5' CAGTCGACTCAGCGGGCCACCTGCAGGAG 3'	*Bgl*II, *Sal*I
pcDNA3-RecQL4-mCer and pcDNA3-RecQL4-mVen	5' CGTGAATTCATGGAGCGGCTGCGGGACGTG 3'<br>5' CACTCGAGGCGGGCCACCTGCAGGAGC 3'	*Eco*RI, *Xho*I
pmCer-RPA32 and pmVen-RPA32	5' CTTGAATTCTGCCACCATGTGGAACAGTGGATTCG 3'<br>5' CGTGGATCCTTATTCTGCATCTGTGGATT 3'	*Eco*RI, *Bam*HI
pcDNA3-RPA32-mCer and pcDNA3-RPA32-mVen	5' CTTGGATCCGCCACCATGTGGAACAGTGGATTCG 3'<br>5' CTTGAATTCTTCTGCATCTGTGGATTT 3'	*Bam*HI, *Eco*RI
pmCer-TopBP1 and pmVen-TopBP1	5' GTCGAGCTCACATGTCCAGAAATGACAAAGAAC 3'<br>5' CGTGGATCCTGCGATTAGTGTACTCTAGG 3'	*Sac*I, *Bam*HI
pcDNA3-TopBP1-mCer and pcDNA3-TopBP1-mVen	5' GACGGATCCATGTCCAGAAATGACAAAGAAC 3'<br>5' CAAGCGGCCGCGAGTGTACTCTAGGTCGTTTG 3'	*Bam*HI, *Not*I
pRSF-Duet-6His-C17orf53	5' GACGAATTCTATGGCGTGCAGTTTGCAG 3'<br>5' CGCGTCGACTCAACTACTGGTCCCACAGAAG 3'	*Eco*RI, *Sal*I

Supplementary Table S2: Protein-ID of HROB homologs shown in Figure 1D

Species	Database	Protein-ID
Alligator mississippiensis	NCBI protein	XP_014464725.1
Callorhinchus milii	NCBI protein	XP_007899727.1
Danio rerio	NCBI protein	XP_689494.2
Eptatretus burger	UniParc	UPI000F35BD7F
Homo sapiens	NCBI protein	NP_076937.2
Latimeria chalumnae	NCBI protein	XP_005992264.1
Mus musculus	NCBI protein	XP_006533103.1
Ornithorhynchus anatinus	NCBI protein	XP_028931947.1
Pelodiscus sinensis	NCBI protein	XP_006125782.1
Phascolarctos cinereus	NCBI protein	XP_020853460.1
Python bivittatus	NCBI protein	XP_025020624.1
Rhinatrema bivittatum	NCBI protein	XP_029428153.1
Taeniopygia guttata	NCBI protein	XP_030111841.1
Xenopus laevis	NCBI protein	XP_018090306.1

Supplementary Table S3: Protein-ID of HROB homologs shown in Supplementary Figure S3

Species	Database	Protein-ID
Amphimedon queenslandica	NCBI protein	XP_019862509.1
Arabidopsis thaliana	NCBI protein	NP_001320641.1
Asparagus officinalis	NCBI protein	XP_020262043.1
Bombyx mori	NCBI protein	XP_004931790.1
Chlamydomonas reinhardtii	NCBI protein	XP_001693214.1
Chytriomyces confervae	NCBI protein	TPX75457.1
Dictyostelium purpureum	NCBI protein	XP_003292044.1
Ectocarpus siliculosus	NCBI protein	CBN75592.1
Hydra vulgaris	NCBI protein	XP_012555373.1
Octopus vulgaris	NCBI protein	XP_029650574.1
Paramicrosporidium saccamoebae	NCBI protein	PJF17609.1
Physcomitrella patens	NCBI protein	XP_024370473.1
Phytophthora parasitica	NCBI protein	ETK91384.1
Plasmodium vivax	NCBI protein	VUZ97071.1
Reticulomyxa filosa	UniProt	X6NW86
Salpingoeca rosetta	NCBI protein	XP_004991073.1
Schistosoma japonicum	NCBI protein	TNN10734.1
Smittium simulii	NCBI protein	PVU95124.1
Sphaeroforma arctica	NCBI protein	XP_014146907.1
Strongylocentrotus purpuratus	NCBI protein	XP_030846213.1
Trichoplax sp. H2	NCBI protein	RDD38568.1
Trichuris trichiura	UniProt	A0A077ZCI7
Trypanosoma cruzi	UniProt	Q4DMJ5

Supplementary Table S4: Quantification of the FRET experiments presented in Figure 2 and Supplementary Figure S11. Data for individual experiments are assembled in Supplementary Tables S5 and S6

	FRET donor	FRET acceptor	FRET^{+} average	FRET^{+} SEM*	n**
**HROB-Cdc45**	Cer-HROB	Cdc45-Ven	2.29%		(n=1)
	HROB-Cer	Cdc45-Ven	14.61%	4.68%	(n=7)
	Cdc45-Cer	Ven-HROB	1.86%	1.01%	(n=4)
	Cdc45-Cer	HROB-Ven	0.49%	0.27%	(n=6)
**HROB-RPA32**	Cer-HROB	Ven-RPA32	0.33%		(n=1)
	Cer-HROB	RPA32-Ven	0.00%		(n=1)
	HROB-Cer	Ven-RPA32	0.00%		(n=1)
	HROB-Cer	RPA32-Ven	2.32%	0.87%	(n=7)
	RPA32-Cer	HROB-Ven	18.78%	10.29%	(n=2)
**HROB-Mcm5**	Cer-HROB	Ven-Mcm5	20.44%	7.58%	(n=2)
	HROB-Cer	Ven-Mcm5	0.62%	0.18%	(n=9)
	HROB-Cer	Mcm5-Ven	15.26%	1.73%	(n=5)
	Cer-Mcm5	Ven-HROB	12.27%	4.18%	(n=4)
	Cer-Mcm5	HROB-Ven	3.07%	1.49%	(n=6)
**HROB-Mcm2**	Cer-HROB	Ven-Mcm2	0.00%		(n=1)
	HROB-Cer	Ven-Mcm2	0.00%	0.00%	(n=3)
	HROB-Cer	Mcm2-Ven	1.10%	0.29%	(n=3)
**HROB-RecQL4**	Cer-HROB	Ven-RecQL4	0.00%		(n=1)
	HROB-Cer	Ven-RecQL4	0.00%	0.00%	(n=3)
	HROB-Cer	RecQL4-Ven	0.00%	0.00%	(n=3)
**HROB-TopBP1**	Cer-HROB	Ven-TopBP1	0.00%		(n=1)
	Cer-HROB	TopBP1-Ven	0.00%		(n=1)
	HROB-Cer	Ven-TopBP1	0.00%	0.00%	(n=3)
	HROB-Cer	TopBP1-Ven	0.00%	0.00%	(n=3)
**Cdc45-Mcm5**	Cdc45-Cer	Ven-Mcm5	0.98%	0.17%	(n=8)
	Cdc45-Cer	Mcm5-Ven	16.16%	3.44%	(n=8)
	Cer-Mcm5	Cdc45-Ven	7.34%	4.60%	(n=8)
	Mcm5-Cer	Cdc45-Ven	38.78%	5.60%	(n=4)
**Cdc45-RecQL4**	Cdc45-Cer	Ven-RecQL4	0.07%	0.03%	(n=7)
	Cdc45-Cer	RecQL4-Ven	5.98%	2.76%	(n=5)
	Cer-RecQL4	Cdc45-Ven	1.75%	1.00%	(n=7)
	RecQL4-Cer	Cdc45-Ven	22.33%	2.76%	(n=9)

*SEM = standard error of the mean

**Number of independent biological replicates

Supplementary Table S5: Overview of the values determined for single cells (cells), Cerulean and Venus double positive cells (Cer⁺ & Ven⁺) and FRET⁺ cells (FRET⁺) in the cytometry FRET assays with HROB fusion proteins (corresponding to Figure 2)

FRET donor	FRET acceptor	replicate	Cells	Cer^+ & Ven^+	$FRET^+$
Cer-HROB	Cdc45-Ven	1	45800	787 (1.72%)	18 (2.29%)
HROB-Cer	Cdc45-Ven	1	24400	2753 (11.28%)	891 (32.36%)
HROB-Cer	Cdc45-Ven	2	17700	2394 (13.53%)	833 (34.80%)
HROB-Cer	Cdc45-Ven	3	27400	1498 (5.47%)	124 (8.28%)
HROB-Cer	Cdc45-Ven	4	38600	1505 (3.90%)	145 (9.63%)
HROB-Cer	Cdc45-Ven	5	88100	1366 (1.55%)	156 (11.42%)
HROB-Cer	Cdc45-Ven	6	6256	371 (5.93%)	15 (4.04%)
HROB-Cer	Cdc45-Ven	7	45700	2636 (5.76%)	46 (1.74%)
Cdc45-Cer	Ven-HROB	1	45400	4540 (10.00%)	92 (2.02%)
Cdc45-Cer	Ven-HROB	2	40400	3409 (8.44%)	174 (5.10%)
Cdc45-Cer	Ven-HROB	3	27500	9922 (36.08%)	28 (0.28%)
Cdc45-Cer	Ven-HROB	4	16500	4157 (25.19%)	1 (0.02%)
Cdc45-Cer	HROB-Ven	1	13000	1593 (12.25%)	0 (0%)
Cdc45-Cer	HROB-Ven	2	24900	1722 (6.92%)	0 (0%)
Cdc45-Cer	HROB-Ven	3	34400	12200 (35.47%)	11 (0.09%)
Cdc45-Cer	HROB-Ven	4	17700	6223 (35.16%)	4 (0.06%)
Cdc45-Cer	HROB-Ven	5	36000	5179 (14.37%)	55 (1.06%)
Cdc45-Cer	HROB-Ven	6	14100	2369 (16.80%)	41 (1.73%)
Cer-HROB	Ven-RPA32	1	54600	302 (0.55%)	1 (0.33%)
Cer-HROB	RPA32-Ven	1	49500	659 (1.33%)	0 (0%)
HROB-Cer	Ven-RPA32	1	23800	1720 (7.23%)	0 (0%)
HROB-Cer	RPA32-Ven	1	17700	4119 (23.27%)	319 (7.74%)
HROB-Cer	RPA32-Ven	2	29000	4693 (16.18%)	125 (2.66%)
HROB-Cer	RPA32-Ven	3	82300	6675 (8.11%)	68 (1.02%)
HROB-Cer	RPA32-Ven	4	40500	3919 (9.68%)	60 (1.53%)
HROB-Cer	RPA32-Ven	5	12000	2463 (20.53%)	20 (0.81%)
HROB-Cer	RPA32-Ven	6	6612	1693 (25.60%)	28 (1.65%)
HROB-Cer	RPA32-Ven	7	44600	2830 (6.35%)	24 (0.85%)
RPA32-Cer	HROB-Ven	1	28000	1840 (6.57%)	2 (0.11%)
RPA32-Cer	HROB-Ven	2	22700	1452 (6.40%)	6 (0.41%)
Cer-HROB	Ven-Mcm2	1	56200	157 (0.28%)	0 (0%)
HROB-Cer	Ven-Mcm2	1	64600	4529 (7.01%)	0 (0%)
HROB-Cer	Ven-Mcm2	2	65200	4646 (7.13%)	0 (0%)
HROB-Cer	Ven-Mcm2	3	45400	1937 (4.27%)	0 (0%)
HROB-Cer	Mcm2-Ven	1	53200	3529 (6.63%)	61 (1.73%)
HROB-Cer	Mcm2-Ven	2	21400	1203 (5.62%)	13 (1.08%)
HROB-Cer	Mcm2-Ven	3	32500	1196 (3.68%)	6 (0.50%)

Supplementary Table S5 continued.

FRET donor	FRET acceptor	replicate	Cells	Cer+ & Ven+	FRET+
Cer-HROB	Ven-Mcm5	1	46200	14 (0.03%)	7 (50.00%)
Cer-HROB	Ven-Mcm5	2	51700	65 (0.13%)	16 (24.61%)
HROB-Cer	Ven-Mcm5	1	26500	1618 (6.11%)	6 (0.37%)
HROB-Cer	Ven-Mcm5	2	69900	2571 (3.68%)	12 (0.47%)
HROB-Cer	Ven-Mcm5	3	92400	5918 (6.40%)	18 (0.30%)
HROB-Cer	Ven-Mcm5	4	96600	5159 (5.34%)	6 (0.12%)
HROB-Cer	Ven-Mcm5	5	73000	4383 (6.00%)	26 (0.59%)
HROB-Cer	Ven-Mcm5	6	41200	2679 (6.50%)	2 (0.07%)
HROB-Cer	Ven-Mcm5	7	91500	3505 (3.83%)	63 (1.80%)
HROB-Cer	Ven-Mcm5	8	43000	2532 (5.89%)	26 (0.59%)
HROB-Cer	Ven-Mcm5	9	90600	3336 (3.68%)	43 (1.29%)
HROB-Cer	Mcm5-Ven	1	26400	1045 (3.96%)	237 (22.68%)
HROB-Cer	Mcm5-Ven	2	96600	5677 (5.88%)	868 (15.29%)
HROB-Cer	Mcm5-Ven	3	81300	4827 (5.94%)	591 (12.24%)
HROB-Cer	Mcm5-Ven	4	60800	2482 (4.08%)	307 (12.37%)
HROB-Cer	Mcm5-Ven	5	40800	2231 (5.47%)	306 (13.72%)
Cer-Mcm5	Ven-HROB	1	31000	919 (2.96%)	5 (0,54%)
Cer-Mcm5	Ven-HROB	2	23500	518 (2.20%)	43 (8.30%)
Cer-Mcm5	Ven-HROB	3	24400	261 (1.07%)	49 (18.77%)
Cer-Mcm5	Ven-HROB	4	27500	247 (0.90%)	53 (21.46%)
Cer-Mcm5	HROB-Ven	1	23200	467 (2.01%)	0 (0%)
Cer-Mcm5	HROB-Ven	2	20600	616 (2.99%)	0 (0%)
Cer-Mcm5	HROB-Ven	3	15900	475 (2.99%)	7 (1.47%)
Cer-Mcm5	HROB-Ven	4	10800	505 (4.68%)	5 (0.99%)
Cer-Mcm5	HROB-Ven	5	38200	134 (0.35%)	13 (9.70%)
Cer-Mcm5	HROB-Ven	6	44700	191 (0.43%)	12 (6.28%)
Cer-HROB	Ven-RecQL4	1	94200	900 (0.96%)	0 (0%)
HROB-Cer	Ven-RecQL4	1	30400	1106 (3.64%)	0 (0%)
HROB-Cer	Ven-RecQL4	2	17300	310 (1.79%)	0 (0%)
HROB-Cer	Ven-RecQL4	3	30300	481 (1.59%)	0 (0%)
HROB-Cer	RecQL4- Ven	1	91600	4272 (4.66%)	0 (0%)
HROB-Cer	RecQL4- Ven	2	37800	1688 (4.47%)	0 (0%)
HROB-Cer	RecQL4- Ven	3	36000	1115 (3.10%)	0 (0%)
Cer-HROB	Ven-TopBP1	1	17500	357 (2.04%)	0 (0%)
Cer-HROB	TopBP1-Ven	1	9961	268 (2.69%)	0 (0%)
HROB-Cer	Ven-TopBP1	1	17300	944 (5.46%)	0 (0%)
HROB-Cer	Ven-TopBP1	2	8827	460 (5.21%)	0 (0%)
HROB-Cer	Ven-TopBP1	3	29200	221 (0.76%)	0 (0%)
HROB-Cer	TopBP1-Ven	1	24100	3911 (16.23%)	0 (0%)
HROB-Cer	TopBP1-Ven	2	2573	293 (11.39%)	0 (0%)
HROB-Cer	TopBP1-Ven	3	5061	804 (15.89%)	0 (0%)

Supplementary Table S6: Overview of the values determined for single cells (cells), Cerulean and Venus double positive cells (Cer$^+$ & Ven$^+$) and FRET$^+$ cells (FRET$^+$) in the cytometry FRET assays with Cdc45 fusion proteins (corresponding to Supplementary Figure 11)

FRET donor	FRET acceptor	replicate	Cells	Cer$^+$ & Ven$^+$	FRET$^+$
Cdc45-Cer	Ven-Mcm5	1	578000	22301 (38.54%)	200 (0.85%)
Cdc45-Cer	Ven-Mcm5	2	54400	20300 (37.32%)	150 (0.74%)
Cdc45-Cer	Ven-Mcm5	3	4630	1427 (30.82%)	17 (1.19%)
Cdc45-Cer	Ven-Mcm5	4	15200	3434 (22.59%)	64 (1.86%)
Cdc45-Cer	Ven-Mcm5	5	31000	13900 (44.84%)	112 (0.81%)
Cdc45-Cer	Ven-Mcm5	6	82900	6814 (8.22%)	4 (0.06%)
Cdc45-Cer	Ven-Mcm5	7	84300	26500 (31.44%)	259 (0.98%)
Cdc45-Cer	Ven-Mcm5	8	82400	29300 (35.56%)	395 (1.35%)
Cdc45-Cer	Mcm5-Ven	1	53900	14500 (26.90%)	1567 (10.81%)
Cdc45-Cer	Mcm5-Ven	2	72400	21700 (29.97%)	2806 (12.93%)
Cdc45-Cer	Mcm5-Ven	3	5268	1096 (20.80%)	408 (37.23%)
Cdc45-Cer	Mcm5-Ven	4	16600	3814 (22.98%)	1051 (27.56%)
Cdc45-Cer	Mcm5-Ven	5	72500	14800 (20.41%)	1431 (9.67%)
Cdc45-Cer	Mcm5-Ven	6	81700	25100 (30.27%)	2660 (10.60%)
Cdc45-Cer	Mcm5-Ven	7	57300	12900 (22.51%)	1371 (10.63%)
Cdc45-Cer	Mcm5-Ven	8	56700	12100 (21.34%)	1194 (9.87%)
Cer-Mcm5	Cdc45-Ven	1	9566	734 (7.67%)	58 (7.90%)
Cer-Mcm5	Cdc45-Ven	2	54700	4791 (8.76%)	113 (2.36%)
Cer-Mcm5	Cdc45-Ven	3	42200	92 (0.22%)	38 (41.30%)
Cer-Mcm5	Cdc45-Ven	4	110000	46 (0.04%)	1 (2.17%)
Cer-Mcm5	Cdc45-Ven	5	74400	1638 (2.20%)	9 (0.55%)
Cer-Mcm5	Cdc45-Ven	6	118000	3102 (2.63%)	8 (0.26%)
Cer-Mcm5	Cdc45-Ven	7	69500	5124 (7.37%)	112 (2.19%)
Cer-Mcm5	Cdc45-Ven	8	86600	4837 (5.59%)	95 (1.96%)
Mcm5-Cer	Cdc45-Ven	1	31200	2108 (6.76%)	1007 (47.77%)
Mcm5-Cer	Cdc45-Ven	2	29500	2458 (8.33%)	1265 (51.46%)
Mcm5-Cer	Cdc45-Ven	3	41100	3177 (7.73%)	774 (24.36%)
Mcm5-Cer	Cdc45-Ven	4	46800	3385 (7.23%)	1067 (31.52%)
Cdc45-Cer	Ven-RecQL4	1	19100	2507 (13.13%)	5 (0.20%)
Cdc45-Cer	Ven-RecQL4	2	25300	7878 (31.14%)	4 (0.05%)
Cdc45-Cer	Ven-RecQL4	3	87000	32700 (37.59%)	7 (0.02%)
Cdc45-Cer	Ven-RecQL4	4	24600	8767 (35.64%)	2 (0.02%)
Cdc45-Cer	Ven-RecQL4	5	19200	7481 (38.96%)	5 (0.07%)
Cdc45-Cer	Ven-RecQL4	6	55100	14700 (26.68%)	17 (0.12%)
Cdc45-Cer	Ven-RecQL4	7	64200	478 (0.74%)	0 (0%)
Cdc45-Cer	RecQL4-Ven	1	3443	792 (23.00%)	139 (17.55%)
Cdc45-Cer	RecQL4-Ven	2	26600	5943 (22.34%)	108 (1.82%)
Cdc45-Cer	RecQL4-Ven	3	14000	3165 (22.61%)	14 (0.44%)
Cdc45-Cer	RecQL4-Ven	4	1961	438 (22.34%)	30 (6.85%)
Cdc45-Cer	RecQL4-Ven	5	1812	276 (15.23%)	9 (3.26%)

Supplementary Table S6 continued.

FRET donor	FRET acceptor	replicate	Cells	Cer^+ & Ven^+	$FRET^+$
Cer-RecQL4	Cdc45-Ven	1	19508	2229 (11.43%)	14 (0.63%)
Cer-RecQL4	Cdc45-Ven	2	16000	5736 (35.85%)	4 (0.07%)
Cer-RecQL4	Cdc45-Ven	3	59500	4911 (8.25%)	28 (0.57%)
Cer-RecQL4	Cdc45-Ven	4	22200	2937 (13.23%)	2 (0.07%)
Cer-RecQL4	Cdc45-Ven	5	14797	2244 (15.17%)	11 (0.49%)
Cer-RecQL4	Cdc45-Ven	6	23200	63 (0.27%)	5 (7.94%)
Cer-RecQL4	Cdc45-Ven	7	14300	40 (0.28%)	1 (2.50%)
RecQL4-Cer	Cdc45-Ven	1	20300	2584 (12.73%)	460 (17.80%)
RecQL4-Cer	Cdc45-Ven	2	14300	834 (5.83%)	236 (28.30%)
RecQL4-Cer	Cdc45-Ven	3	355	89 (25.07%)	21 (23.60%)
RecQL4-Cer	Cdc45-Ven	4	5677	711 (12.52%)	134 (18.85%)
RecQL4-Cer	Cdc45-Ven	5	2982	383 (12.84%)	113 (29.50%)
RecQL4-Cer	Cdc45-Ven	6	2581	567 (21.97%)	162 (28.57%)
RecQL4-Cer	Cdc45-Ven	7	5295	838 (15.83%)	99 (11.81%)
RecQL4-Cer	Cdc45-Ven	8	17600	1918 (10.90%)	157 (8.19%)
RecQL4-Cer	Cdc45-Ven	9	4757	660 (13.87%)	227 (34.39%)

Supplementary References

140. Spatafora, J.W.; Aime, M.C.; Grigoriev, I.V.; Martin, F.; Stajich, J.E.; Blackwell, M. The Fungal Tree of Life: From Molecular Systematics to Genome-Scale Phylogenies. In *The Fungal Kingdom*; Wiley: Hoboken, NJ, USA, 2017. https://doi.org/10.1128/9781555819583.ch1.
141. Pennisi, E. Drafting a Tree. *Science* **2003**, *300*, 1694.
142. Uhlen, M.; Zhang, C.; Lee, S.; Sjöstedt, E.; Fagerberg, L.; Bidkhori, G.; Benfeitas, R.; Arif, M.; Liu, Z.; Edfors, F.; et al. A Pathology Atlas of the Human Cancer Transcriptome. *Science* **2017**, *357*, eaan2507.
